# Integrated physiological and transcriptomic analysis uncovers the mechanism of moderate nitrogen application on promoting the growth and (-)-borneol accumulation of *Blumea balsamifera*

**DOI:** 10.3389/fpls.2024.1531932

**Published:** 2025-01-31

**Authors:** Yuan Yuan, Wei-Jie Tang, Jia-Yuan Cao, Ke Zhong, Ze-Jun Mo, Ying Zhou, Yu-Xin Pang

**Affiliations:** ^1^ School of Biosciences and Biopharmaceutics, Guangdong Pharmaceutical University, Guangzhou, China; ^2^ College of Pharmaceutical Sciences, Guizhou University of Traditional Chinese Medicine, Guiyang, China; ^3^ Research Center for Ecological Planting Technology of Traditional Chinese and Ethnic Medicines, Guizhou University of Traditional Chinese Medicine, Guiyang, China; ^4^ Yunfu Traditional Chinese Medicine Resources and Germplasm Resources Database Management Center, the Government of Yunfu City, Yunfu, China

**Keywords:** *Blumea balsamifera*, nitrogen, transcriptome, growth, (-)-borneol, terpenoid

## Abstract

**Introduction:**

*Blumea balsamifera*, a half-woody plant belonging to the Asteraceae family, is valued as both a medicinal and industrial crop primarily for its phytochemical component, (-)-borneol. Nitrogen (N) is essential for regulating the growth of *B. balsamifera* and the biosynthesis of (-)-borneol; however, the molecular mechanisms by which N influences these processes remain inadequately understood. This study aimed to elucidate the effects of N on growth and (-)-borneol synthesis at the molecular level.

**Methods:**

A field experiment was conducted in which *B. balsamifera* plants were fertilized with three different nitrogen regimes: 0 kg N ha^-1^ (control, CK), 150 kg N ha^-1^ (N1 treatment), and 300 kg N ha^-1^ (N2 treatment). Physiological and biochemical assessments were performed to evaluate the growth and metabolic responses of the plants under these varying N conditions. Additionally, transcriptome sequencing of leaves of *B. balsamifera* was conducted to elucidate the underlying molecular mechanisms involved.

**Results and discussion:**

The results indicated that both the N1 and N2 treatments significantly promoted the growth of *B. balsamifera*, with the 150 kg N ha^-1^ treatment (N1) resulting in the most favorable effects. Under the N1 treatment, the leaves harvested in October, November, and December exhibited the highest accumulation of (-)-borneol, with yields of 782 mg plant^-1^, 1102 mg plant^-1^, and 1774 mg plant^-1^, respectively, which were significantly different from those observed in the CK and N2 treatments. Comparative transcriptome analysis revealed a total of 6,714 differentially expressed genes (DEGs). Notably, several DEGs associated with auxin signaling and N metabolism were upregulated in the N1 and N2 treatments. In contrast, many DEGs related to carbohydrate metabolism, terpenoid backbone biosynthesis, monoterpenoid biosynthesis, and flavonoid biosynthesis were significantly upregulated in the CK treatment. Moreover, potential transcription factors (TFs) that may link N nutrition with the synthesis of medicinal components were identified. Our study demonstrates that N can enhance the accumulation of (-)-borneol in *B. balsamifera* when applied in appropriate quantities. These findings provide a comprehensive understanding of the relationship between N nutrition and (-)-borneol yield in *B. balsamifera*, offering valuable insights for future cultivation practices.

## 1 Introduction

*Blumea balsamifera*, also known as Ainaxiang in China, is a perennial woody herb in the Asteraceae family and is distributed across several countries in Southeast Asia and South Asia, notably China, Pakistan, India, Myanmar, Thailand, Malaysia, Indonesia, and the Philippines. In China, it is distributed mainly in the provinces of Guizhou, Guangxi, Yunnan, Hainan, and Guangdong (Guan et al., 2024b). The initial documentation of this plant occurred in the year 652 within the text “Bei Ji Qian Jin Yao Fang,” authored by Sun Simiao (Pang et al., 2014). Traditional Chinese medicine, in its early forms, employs either the entire plant or its leaves to treat various illnesses, including beriberi, dermatitis, eczema, lumbago, menorrhagia, rheumatism, and skin injuries, and as insect repellents. Currently, it is also used as a medicinal and industrial crop for extracting the main phytochemical component, (-)-borneol (Guan et al., 2024a), which is known commercially as borneol flake and is an important secondary metabolite and the main active medicinal component in *B. balsamifera* (Yuan et al., 2016b). Since 2010, *B. balsamifera* has been recognized as the sole plant source for borneol flakes in the Pharmacopoeia of the People’s Republic of China (Pang et al., 2014). Modern pharmacological studies have confirmed that (-)-borneol, a bicyclic monoterpene compound, has anti-inflammatory, antioxidant, analgesic, drug absorption-promoting, rejuvenation and other effects (Guan et al., 2016). The refinement of borneol flakes yields blumea oil as a byproduct, renowned in pharmaceuticals, cosmetics, fragrances, and fine chemicals for its distinctive aroma (Yu et al., 2017). Flavonoids, which mainly include quercetin, pinocembrin chalcone, pinocembrin, and blumeatin, are abundant in the plant residue after (-)-borneol distillation (Xia et al., 2016). Our previous pharmacological study confirmed that flavonoids are the main active components in *B. balsamifera* that contribute to excisional wound healing (Pang et al., 2017). Furthermore, various products that use *B. balsamifera* as a raw material provide great economic and social benefits, resulting in increasing demand for (-)-borneol and flavonoids (Yuan et al., 2016b). However, in the cultivation of *B. balsamifera*, the yield of (-)-borneol is only 45~60 kg ha^-1^, which is far from meeting the market demand. Therefore, increasing the yield of (-)-borneol per plant is an urgent need.

Nitrogen (N) is one of the most important nutrients for plants and is one of the primary environmental factors influencing plant growth, physiology, and metabolism (Saloner and Bernstein, 2021). Moderate N application can promote the increase in biomass and secondary metabolite accumulation of many medicinal plants, such as *Stevia rebaudiana* (Sun et al., 2021), *Panax notoginseng* (Cun et al., 2024) and *Robinia pseudoacacia* (Li et al., 2024). Moreover, two experiments have been conducted to explore the influences of various N regimes on the yield of *B. balsamifera* and its secondary metabolism (Lan et al., 2017; Gu et al., 2018). Nevertheless, as the experimental conditions and plant materials of the two studies above were different, the results of the two studies were inconsistent. Our previous study additionally revealed that the (-)-borneol and total flavonoid contents and accumulation in *B. balsamifera* leaves were significantly greater than those in other organs (Huang et al., 2016; Yuan et al., 2016b). Therefore, N fertilizer management strategies that optimize the yield and medicinal component of *B. balsamifera* leaves are needed.

Although N plays an important role in regulating *B. balsamifera* growth and (-)-borneol/total flavonoid synthesis, studies of *B. balsamifera* leaves under different N regimes at the molecular level are rare. Recently, with the development of transcriptomic technology, effective tools have become available for identifying key target genes and understanding physiological and transcriptional processes (Sun et al., 2021). For example, a transcriptome study in hybrid paper mulberry illustrated the transcription mechanism that explains the coordinated regulation of morphological, physiological and other related metabolic strategies in response to different N regimes (Ni et al., 2020). Moreover, through transcriptome analysis, key genes related to (-)-borneol synthesis have been screened in *B. balsamifera* leaves treated with varying concentrations of methyl jasmonate (MEJA) (Guan et al., 2024a). In addition, a more recent study characterized key genes that regulate the total flavonoid content in *B. balsamifera* leaves under exogenous MeJA treatment (Guan et al., 2024b). However, transcriptomic analysis has not yet been used to determine the mechanism of N-mediated control of *B. balsamifera* growth, (-)-borneol synthesis and flavonoid synthesis. Here, a field experiment was conducted to investigate the effects of different N regimes on growth parameters, N content and accumulation, and (-)-borneol/total flavonoid content and accumulation in *B. balsamifera* leaves. RNA sequencing (RNA-Seq) analysis of *B. balsamifera* leaf tissue was applied to reveal differences in the molecular mechanisms involved in the response to different N regimes. Some candidate genes associated with (-)-borneol/total flavonoid synthesis, plant hormone signal transduction, N metabolism and carbohydrate metabolism were identified to provide valuable resources for understanding the major transcriptomic regulatory pathways in the leaves of *B. balsamifera* in response to different N regimens.

## 2 Materials and methods

### 2.1 Experimental conditions and plant material

The field experiment was conducted from October–December 2023 in Duanqiao township (25°50’ N, 105°41’ E; altitude 610 m), Guanlin County, Guizhou Province, China. The soil at the testing locations was sandy loam. The chemical properties of the surface soil (0–20 cm) included a pH of 7.18, organic matter content of 35.07 g kg^-1^, total N content of 1.51 g kg^-1^, NO_3_^–^N content of 100.60 mg kg^-1^, NH_4_^+^-N content of 13.68 mg kg^-1^, Olsen P content of 37.45 mg kg^-1^, and available K content of 26.50 mg kg^-1^. The monthly mean temperature and total precipitation during the *B. balsamifera* growing season from June to December 2023 are shown in **Supplementary Figure S1**. The experimental *B. balsamifera* plants were two-year-old bare root seedlings (approximately 30 cm long) collected from Hongshuihe township (25°60’ N, 106°33’ E; altitude 566 m), Luodian County, Guizhou Province, China.

The seedlings were planted at a spacing of 60 cm×60 cm. After 3 weeks of growth, the healthy plants were divided into three treatment groups. We defined the control and two nitrogen (N) treatments as follows: CK, treatment with no N fertilizer; N1, treatment with 150 kg N ha^-1^ (moderate N); and N2, treatment with 300 kg N ha^-1^ (high N) (Huang et al., 2016). N fertilizers in the form of urea (46% N) (Jiangsu Hengsheng Chemical Co., LTD, China) were applied after 4 weeks and 8 weeks of growth at a ratio of 50%:50%. Phosphorus fertilizer in the form of calcium magnesium phosphate (200 kg P_2_O_5_ ha^-1^) (12% P_2_O_5_) (Hubei Golden Pearl Chemical Co., LTD, China) and potassium fertilizer in the form of potassium sulfate (200 kg K_2_O ha^-1^) (52% K_2_O) (Hebei Sanfu Silicon industry Co., LTD, China) were applied after 4 weeks of growth in all the treatments. The experiment was arranged in a completely randomized block design with three replicates. Each plot was 20 m^2^ (2 m width × 10 m length) long and was divided equally to facilitate the planting of the *B. balsamifera* seedlings.

The transcriptome experiment was performed 48 h after the second N application. The leaf of one plant (selecting the 9th leaf counting down from the top) in each plot (three biological replicates for each treatment) were harvested, quickly transferred to liquid N_2_ and stored at -80°C until subsequent analyses.

### 2.2 Sampling and processing

The growth parameters, including the plant height, stem diameter, branch number, leaf number, number of bare root seedlings and leaf soil plant analysis development (SPAD) value, of five plants in each plot were determined in October, November and December, respectively, following the methods of (Gu et al., 2018). Briefly, the plant height was the distance from the ground to the top of the plant; the stem diameter was the stem thickness 5~10 cm above the ground measured with Vernier calipers; the branch number was the number of available primary branches per plant; the leaf number was the total number of leaves per plant; the number of bare root seedlings was the number of shoots that emerged within a 30 cm radius centered on the plant; and the leaf SPAD value was the average SPAD value of the sixth leaf of the plant, which was counted from top to bottom and measured six times with SPAD-502 Plus (Konica Minolta, INC, Japan).

During sampling, five plants with uniform growth per plot in each treatment were cut at the stem base in October, November and December, and the ground parts were divided into stems and leaves. The fresh leaf weight was measured via a one percent electronic balance. Following shading in the laboratory, the leaves were dried at 39°C until a stable weight was reached over 24 hours. The samples were subsequently ground into a fine powder (60-mesh sieve) after the dry weight was determined and then stored in zip-lock bags. The dried leaf samples were digested via a concentrated mixture of H_2_SO_4_ and H_2_O_2_ to determine the N concentration through the micro-Kjeldahl procedure (Wei et al., 2020).

### 2.3 Determination of leaf (-)-borneol and leaf total flavonoids

Gas chromatography (GC) was used to determine the (-)-borneol content of *B. balsamifera* leaves grown under different N treatments for different durations according to our previous method (Yu et al., 2017) with some modifications. The (-)-borneol accumulation (mg plant^-1^) was calculated as (-)-borneol content (mg g^-1^) × dry weight (g plant^-1^).

The total flavonoid contents in dry leaf samples were determined via a UV−Vis spectrophotometer at a maximum absorbance wavelength of 510 nm following our previous method (Yu et al., 2017) with some modifications. The total flavonoid accumulation (mg plant^-1^) was calculated as the total flavonoid content (mg g^-1^) × dry weight (g plant^-1^).

### 2.4 RNA isolation, cDNA library construction and Illumina deep sequencing

For the CK, N1, and N2 treatments, leaf samples were collected, with nine samples in total (three replicates per treatment), and stored at -80°C. RNA extraction was then conducted. Following mRNA isolation, fragment interruption, cDNA synthesis, adapter addition, PCR amplification, and comparative high-throughput RNA sequencing (RNA-Seq), the procedures were carried out by Shanghai Majorbio Biopharm Biotechnology Co., Ltd. (Shanghai, China). In brief, TRIzol^®^ Reagent (Qiagen, Germany) was used to extract total RNA from the samples. Subsequently, The concentration and quality of RNA were assessed with a NanoDrop2000 spectrophotometer (Thermo Scientific, USA). The sequencing libraries were constructed solely from high-quality RNA samples meeting the following criteria: OD260/280 = 1.8~2.2, OD260/230≥2.0, RIN≥6.5, 28S:18S≥1.0, and >1 μg. The remaining RNA was carefully stored for later use (quantitative real-time polymerase chain reaction (qRT−PCR)). An mRNA-Seq library (strand-specific) was constructed. Afterward, the library preparations were sequenced on an Illumina NovaSeq 6000 platform (Illumina, San Diego, USA).

### 2.5 Bioinformatics analysis

The initial raw paired-end reads underwent trimming and quality control via fastp with the default parameters (Chen et al., 2018). The clean data from the nine samples were subsequently employed for *de novo* assembly via Trinity (Grabherr et al., 2011). To increase the assembly quality, all the assembled sequences were subjected to CD-Hit and transrating.

For annotation information, comparisons between unigenes and public databases (Altschul et al., 1997), such as the National Center for Biotechnology Information (NCBI), nonredundant protein sequence database (Nr), evolutionary genealogy of Genes: Nonsupervised Orthologous Groups (eggNOG), Swiss-Prot protein sequence data (SwissProt), protein family analysis and modeling (Pfam), Gene Ontology (GO) and Kyoto Encyclopedia of Genes and Genomes Pathway Database (KEGG), were performed via BLAST (E value < 10^-5^).

iTAK 1.2 software (Zheng et al., 2016) was used to identify transcription factors (TFs) in *B. balsamifera*. To identify differentially expressed genes (DEGs) between any two samples, transcript levels were determined via the transcripts per million reads (TPM) approach. Gene abundances were quantified through RNA-Seq by expectation maximization (RSEM) (Li and Dewey, 2011). Significantly differentially expressed genes (DEGs) were defined as those exhibiting |log_2_FC|≥1 and a false discovery rate (FDR) ≤ 0.05, as determined by DESeq2 (Love et al., 2014). Analysis of correlations among RNA-Seq samples was conducted via principal component analysis (PCA) via MetaboAnalyst 4.0 (Chong et al., 2018). Additionally, GO enrichment analysis and KEGG enrichment analysis were performed via Goatools (*p* value < 0.01) and KOBAS (*p* value < 0.01) (Xie et al., 2011), respectively.

### 2.6 Quantitative RT−PCR analysis

To validate the gene expression patterns identified through Illumina sequencing, quantitative reverse transcription polymerase chain reaction (qRT−PCR) was conducted on 15 selected genes, utilizing total RNA extracted from three biological replicates for each treatment. Gene-specific primer pairs were designed on the basis of the target gene sequences via Primer Premier 5.0 software (**Supplementary Table S1**). First-strand cDNAs were synthesized from 3 µL of total RNA via a Bio-Rad C100 Touch™ Thermal Cycler (Bio-Rad Laboratories, Hercules, CA, USA). The real-time PCR analysis included three independent biological replicates and three technical replicates for each treatment. Each qRT−PCR was performed in a total volume of 20 µL, comprising 10 µL of 2 × SP qPCR mix, 1 µL each of forward and reverse primers, 1 µL of 1:10 diluted cDNA, and 7 µL of double-distilled water. The PCR conditions consisted of an initial denaturation step at 94°C for 20 seconds, followed by 40 cycles of denaturation at 94°C for 10 seconds and annealing/extension at 60°C for 20 seconds. The reactions were carried out using a Bio-Rad CFX96™ Real-Time PCR system (Bio-Rad Laboratories, Hercules, CA, USA). To normalize expression levels, a control primer specific to the *B. balsamifera 18S rRNA* gene (Fan et al., 2023) (**Supplementary Table S1**) was used, and relative expression levels were calculated via the 2^−ΔΔCt^ method (Shen et al., 2024).

### 2.7 Statistical analysis

The data were collated via Excel 2020 (Microsoft Corporation, Redmond, WA, USA). Significant differences among the treatments were evaluated via one-way analysis of variance (ANOVA) conducted with SPSS 26.0. (SPSS Inc., Chicago, IL, USA). Differences were considered significant at *p* < 0.05. Principal component analysis (PCA) was conducted via the prCOMP function in R (www.r-project.org). Heatmaps and Venn diagrams were constructed via TBtools (Chen et al., 2023a), whereas other figures were generated via Origin 2021 and SigmaPlot 12.5.

## 3 Results

### 3.1 Phenotype, biomass and physiology are affected by nitrogen

**Figure 1:**
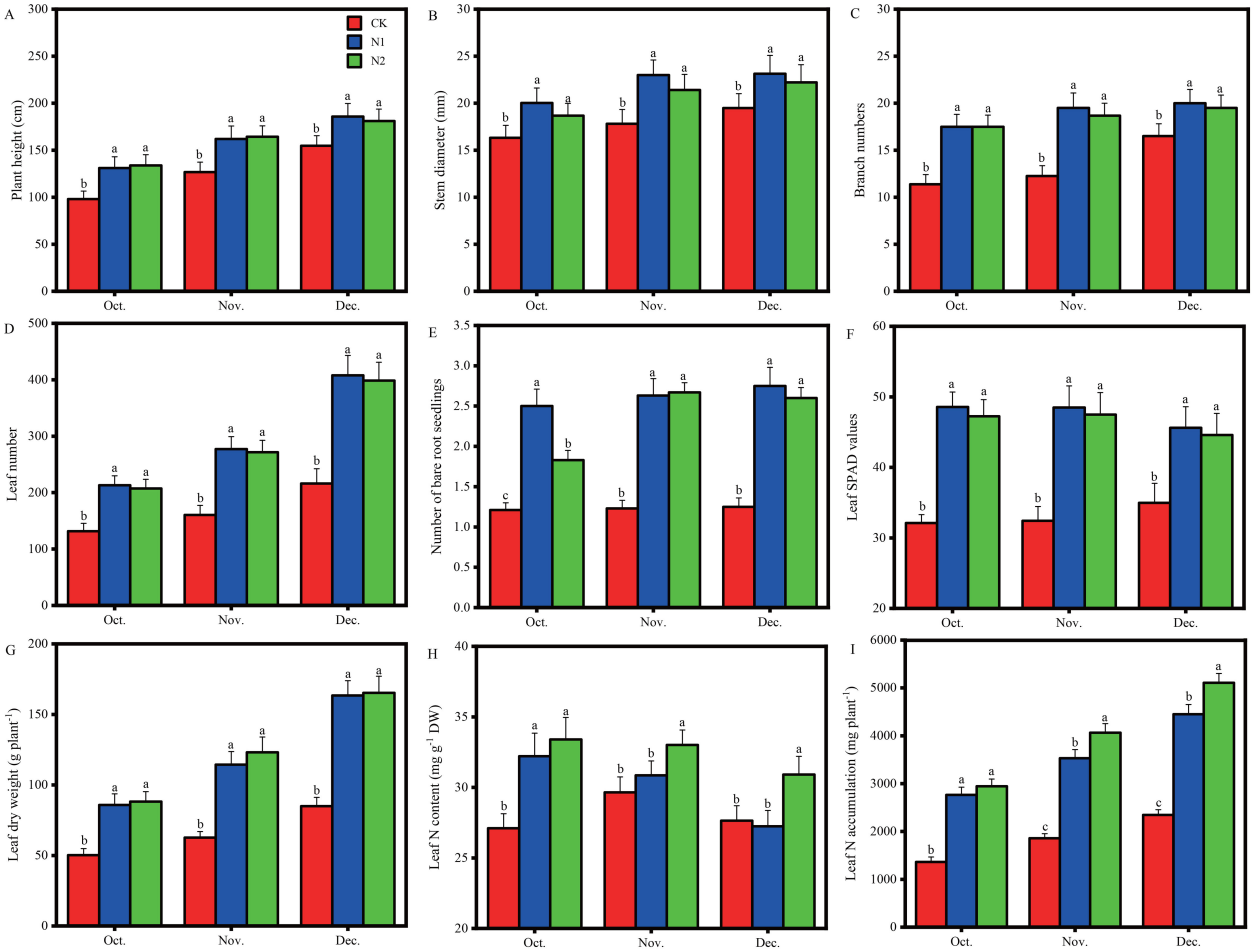
Effects of different N regimes on the phenotype, biomass and physiology of *B balsamifera* in October, November and December. **(A)** Plant height. **(B)** Stem diameter. **(C)** Branch number. **(D)** Leaf number. **(E)** Number of bare root-bearing seedlings. **(F)** Leaf SPAD values. **(G)** Leaf dry weight. **(H)** Leaf N content. **(I)** Leaf N accumulation. The distinct letters above the histogram bars denote statistically significant differences among the treatments within the same month (*p* < 0.05). The vertical bars represent the standard deviation (SD) (n = 5).

The months of October, November and December were suitable for harvesting *B. balsamifera*, and it was generally believed that a higher biomass and medicinal component content could be obtained by harvesting in November (Huang et al., 2016). The growth phenotype, biomass and physiology of *B. balsamifera* were significantly affected by the N regime in different months (**Figure 1**). Overall, the two N application treatments considerably promoted the growth of *B. balsamifera* in October, November and December. The plant height (**Figure 1A**), stem diameter (**Figure 1B**), branch number (**Figure 1C**), leaf number (**Figure 1D**) and leaf SPAD values (**Figure 1F**) in the N1 and N2 treatments were greater than those in the CK treatment in October, November and December, and there were no differences between the N1 and N2 treatments. The number of bare root-bearing seedlings (**Figure 1E**) was greatest in the N1 treatment and lowest in the CK treatment in October, and in the N1 and N2 treatments, it was greater than that in the CK treatment in November and December, and there were no differences between the N1 and N2 treatments.

Compared with the CK treatment, the N1 and N2 treatments greatly increased leaf dry weight by 70.72% and 75.47% in October, 82.52% and 96.38% in November, and 92.39% and 94.64% in December, respectively (**Figure 1G**); significantly increased the leaf N content by 18.86% and 23.25% in October; notably, the N2 treatment increased the leaf N content by 11.33% and 11.83% in November and December, respectively (**Figure 1H**); and remarkably promoted leaf N accumulation by 202.94% and 216.30% in October, 189.89% and 218.61% in November, and 189.65% and 217.68% in December, respectively (**Figure 1I**).

### 3.2 (-)-Borneol and total flavonoids in leaves

**Figure 2:**
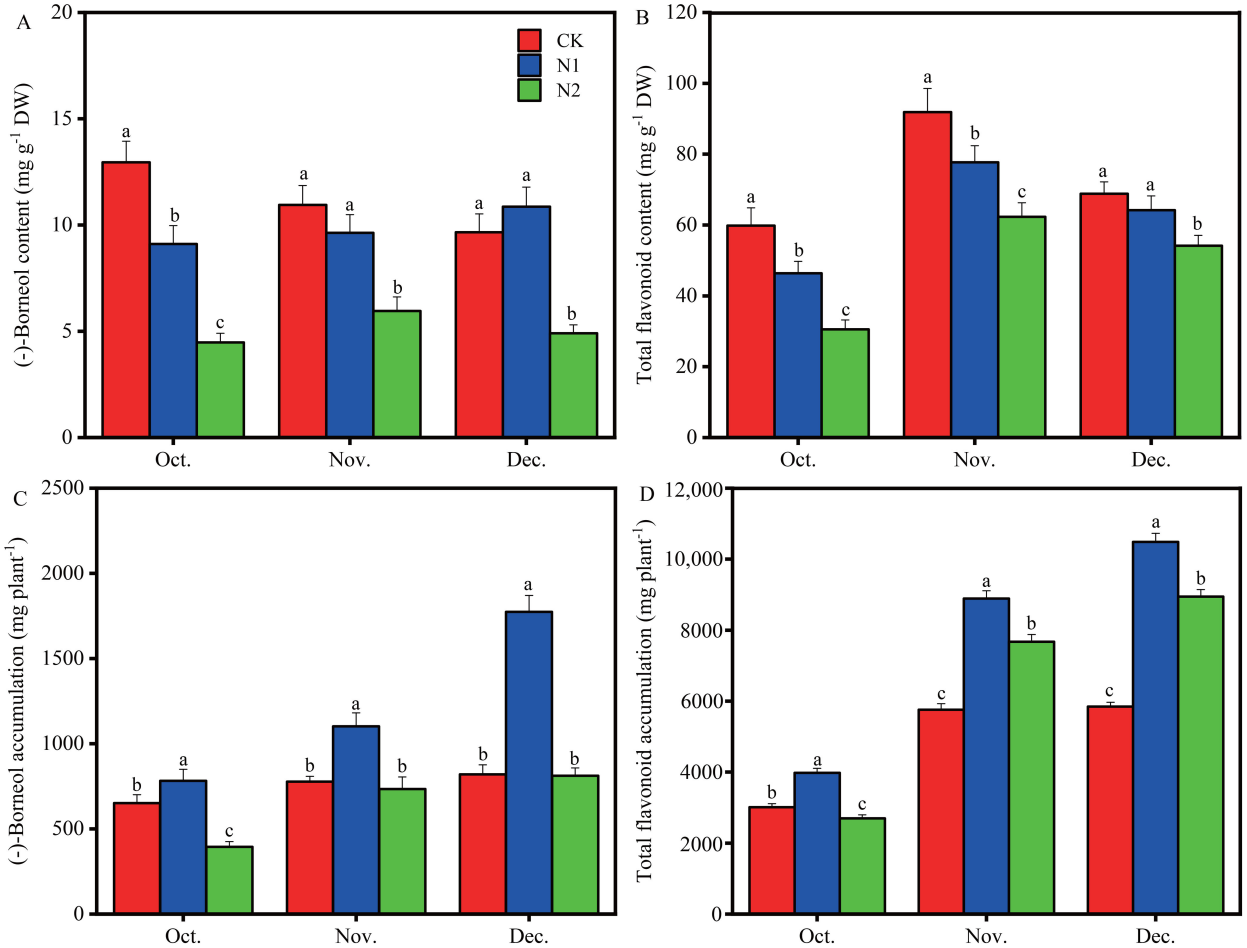
Effects of different N supply levels on the (-)-borneol content **(A)**, total flavonoid content **(B)**, (-)-borneol accumulation **(C)** and total flavonoid accumulation **(D)** of *B balsamifera* leaves in October, November and December. Different letters at the top of the histogram bars denote significant differences among treatments for the same month (*p* < 0.05). The vertical bars indicate the standard deviation (SD) (n = 5).

(-)-Borneol and total flavonoids are the two most important medicinal components of *B. balsamifera* plants and are strongly affected by the N regime (**Figure 2**). Compared with the CK treatment, the N1 and N2 treatments decreased the leaf (-)-borneol content by approximately 29.65% and 65.41%, respectively, in October. The leaf (-)-borneol content was higher in the CK and N1 treatments than in the N2 treatment in November and December (**Figure 2A**), and there were no significant differences between the N1 and CK treatments. Compared with the CK, the N1 and N2 treatments reduced the leaf total flavonoid content by 22.52% and 48.96%, respectively, in October and 15.43% and 32.16% in November. The leaf total flavonoid content was greater in the CK and N1 treatments than in the N2 treatment in December (**Figure 2B**).

Leaf (-)-borneol accumulation was greater in the N1 treatment than in the CK and N2 treatments in October, November and December (**Figure 2C**), at 782 mg plant^-1^, 1102 mg plant^-1^ and 1774 mg plant^-1,^respectively. Compared with the CK and N2 treatments, the N1 treatment remarkably promoted the leaf (-)-borneol accumulation by 120.12% and 197.97% in October, 141.83% and 150.14% in November, and 216.34% and 218.47% in December, respectively (**Figure 2C**). Leaf (-)-borneol accumulation in the CK treatment was greater than that in the N2 treatment in October, whereas there were no significant differences between the CK and N2 treatments in November and December (**Figure 2C**). Leaf total flavonoid accumulation was highest in the N1 treatment in October, November and December and lowest in the N2 treatment in October, whereas it was greatest in the CK treatment in November and December (**Figure 2D**).

### 3.3 Transcriptome sequencing and annotation

After removing sequencing adapters and low-quality data, we obtained 48,252,899, 49,999,671 and 48,541,803 clean reads in the CK, N1 and N2 treatments, respectively, on average, corresponding to approximately 7.04, 7.29 and 7.14 GB of data, respectively. The GC content of each of the transcriptomes ranged from 44.82–45.77%. The values of Q30 were between 93.12 and 93.58% (**Supplementary Table S2**). These results suggested that the RNA sequencing data used in the present study were highly reliable for *de novo* assembly and expression analysis. A collective count of 83,825 unigenes was identified across all the samples (**Supplementary Table S3**). We found that a BLAST+-based homology search for *B. balsamifera* resulted in the annotation of 35,418 unigenes (42.25%) by comparing the unigenes with information from six databases (**Supplementary Table S3**). Among the 83,825 unigenes, 35,112 unigenes (41.89%) were annotated in the Nr database; 27,812 unigenes (33.18%) were annotated within the eggNOG database; 23,363 unigenes (27.87%) were annotated from the Swiss-Prot database; 21,682 unigenes (25.87%) were annotated via the Pfam database; 27,816 unigenes (33.18%) were annotated through the GO database; and 11,799 unigenes (14.08%) were annotated within the KEGG database.

### 3.4 Dynamic transcriptome changes in *Blumea balsamifera* treated with different nitrogen regimes

**Figure 3:**
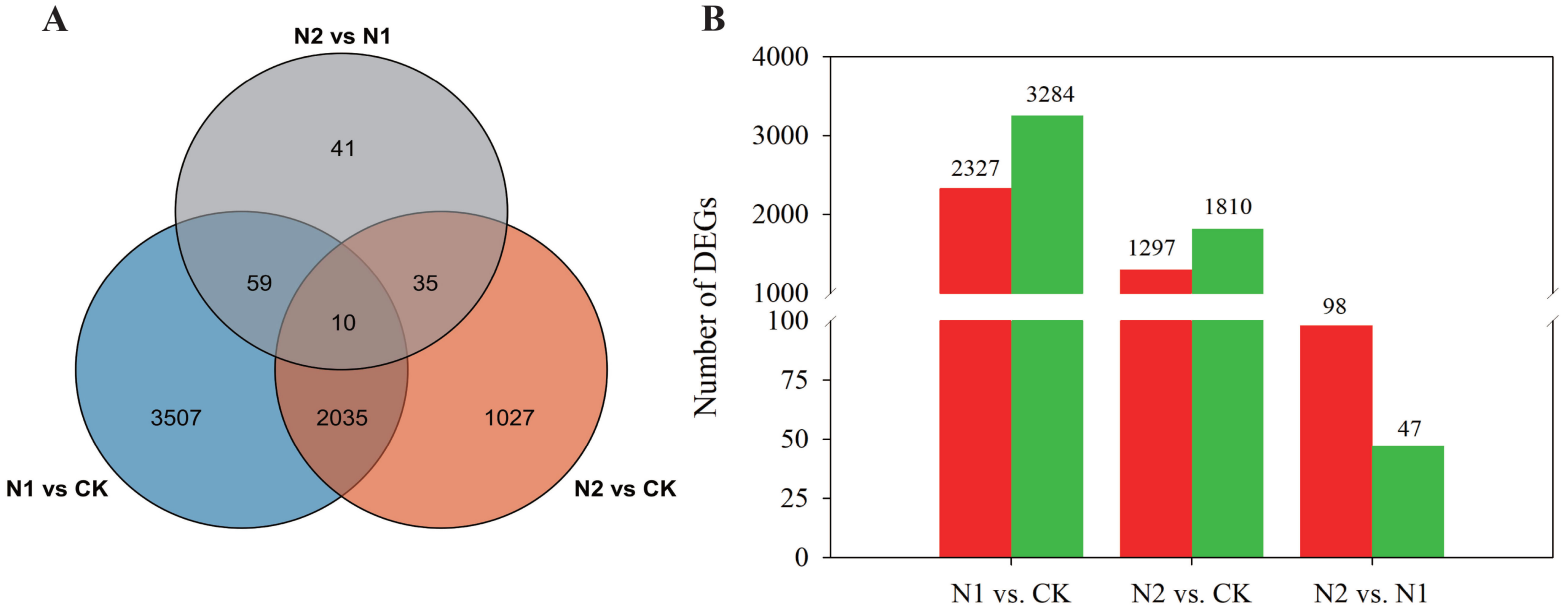
**(A)** A Venn diagram illustrating overlaps among differentially expressed genes (DEGs) in different comparison groups, i.e., N1 vs. CK, N2 vs. CK, and N1 vs. N2. **(B)** Bar chart showing up- and downregulated DEGs in the three comparison groups. The red column displays upregulated DEGs, whereas the green column presents downregulated DEGs.

To evaluate the variability among the RNA-Seq experiments, principal component analysis (PCA) was conducted. The analysis included three biological replicates for each treatment, which were distinctly grouped in the PCA plot. Notably, the three N regimes were clearly separated from one another, as illustrated in **Supplementary Figure S2**. Moreover, using the nine samples, we constructed three comparison groups, N1 vs. CK, N2 vs. CK, and N2 vs. N1, and 6714 unigenes were identified as significantly differentially expressed genes (DEGs) in the three comparison groups, which contained 5611, 3107, and 145 DEGs (**Figure 3A**), respectively. A comparison between the N1 and CK treatments revealed 2327 upregulated and 3264 downregulated DEGs in *B. balsamifera* leaves (**Figure 3B**). A total of 1297 upregulated and 1810 downregulated DEGs were found between the N2 and CK treatments. Furthermore, compared with the N1 treatment, the N2 treatment resulted in only 98 upregulated and 47 downregulated DEGs.

Using the nine samples, we established three comparison groups: N1 vs. CK, N2 vs. CK, and N2 vs. N1. A total of 6,714 unigenes were identified as significantly differentially expressed genes (DEGs) across these comparisons, comprising 5,611 DEGs for N1 vs. CK, 3,107 DEGs for N2 vs. CK, and 145 DEGs for N2 vs. N1 (**Figure 3A**). Specifically, the comparison between the N1 and CK treatments revealed 2,327 upregulated and 3,264 downregulated DEGs in the leaves of *B. balsamifera* (**Figure 3B**). In the N2 vs. CK comparison, we identified 1,297 upregulated and 1,810 downregulated DEGs. Furthermore, when the N2 treatment was compared with the N1 treatment, only 98 DEGs were upregulated, whereas 47 DEGs were downregulated.

A GO enrichment analysis was conducted to categorize all differentially expressed genes (DEGs) into the relevant classifications of “biological process”, “molecular function”, and “cellular component”. The GO terms in each category were sorted from lowest to highest by *p* values (**Supplementary Figure S3**). The top 20 enriched GO terms for the N1 vs. CK comparison are displayed in **Supplementary Figure S3A**. Among these terms, ‘hydrocarbon metabolic process (GO: 0120252)’, ‘terpene biosynthetic process (GO: 0046246)’ and ‘cell wall organization or biogenesis (GO: 0071554)’ belonging to the ‘biological process’ category were the three most enriched GO terms. The top 20 significantly enriched GO terms between the N2 and CK treatments, shown in **Supplementary Figure S3B**, included 11, 1, and 8 subcategories in the ‘biological process’, ‘molecular function’, and ‘cellular component’ categories, respectively. The top 20 significantly enriched GO terms between the N2 and N1 treatments shown in **Supplementary Figure S3C** included 15 and 5 subcategories in the ‘biological process’ and ‘molecular function’, respectively.

**Figure 4:**
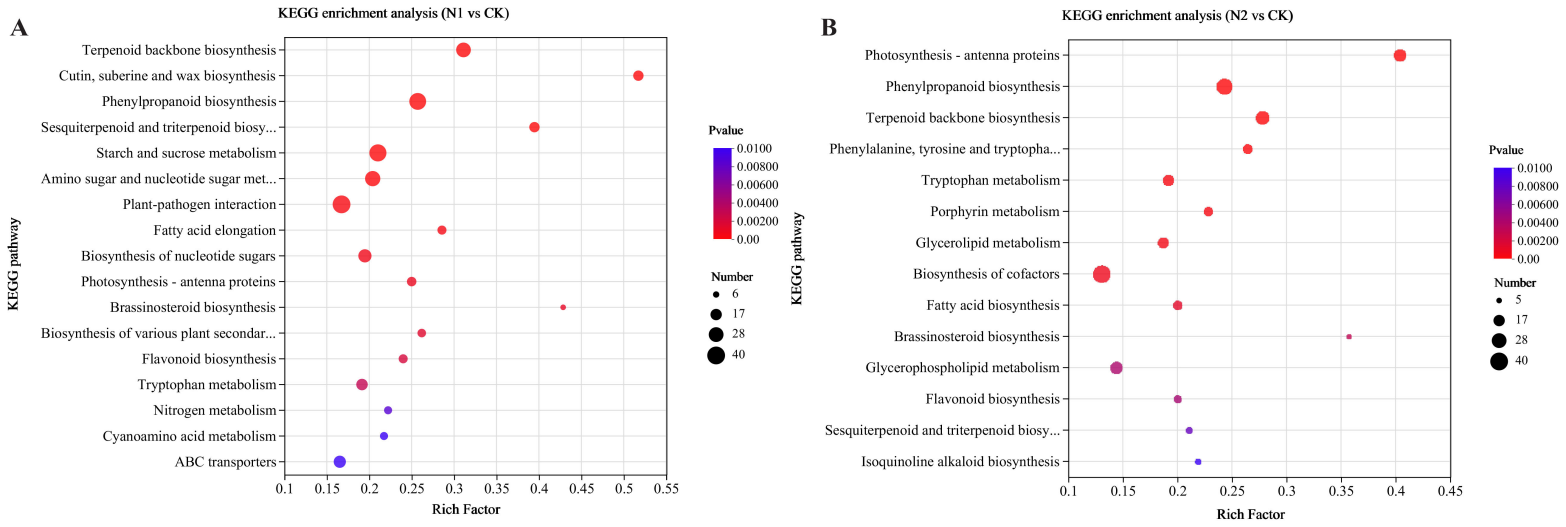
KEGG enrichment analysis of all the DEGs under different N regimens. The most enriched KEGG pathways in the N1 vs. CK **(A)** and N2 vs. CK **(B)** comparisons are presented. The horizontal axis denotes the enrichment factor, with the vertical axis indicating the pathway name. Number: number of DEGs; Padjust: *p* value < 0.01.

Additionally, enrichment analysis via the Kyoto Encyclopedia of Genes and Genomes (KEGG) database was performed to explore metabolic pathways. Scatter plots illustrating all significantly enriched KEGG pathways are depicted in **Figures 4A, B**. There were a total of 17, 14, and 5 significantly enriched KEGG pathways for the N1 vs. CK, N2 vs. CK, and N2 vs. N1 comparisons, respectively. The DEGs in the N1 vs. CK comparison (**Figure 4A**) were enriched mainly in terpenoid backbone biosynthesis, ‘cutin, suberin and wax biosynthesis’ and phenylpropanoid biosynthesis, whereas the DEGs in the N2 vs. CK comparison (**Figure 4B**) were enriched mainly in photosynthesis-antenna proteins, phenylpropanoid biosynthesis and terpenoid backbone biosynthesis. In the N2 vs. N1 comparison (**Supplementary Figure S4**), vitamin B6 metabolism and biosynthesis of cofactors were more enriched in DEGs. Interestingly, terpenoid backbone biosynthesis and flavonoid biosynthesis were significantly enriched in both the N1 vs. CK and N2 vs. CK comparisons, and N metabolism was significantly enriched in the N1 vs. CK comparison.

### 3.5 DEGs involved in plant hormone signal transduction

Phytohormones are essential for how plants adjust to N environments, and the primary hormones involved in managing plant reactions to different N regimes include auxin, cytokinin, gibberellin, abscisic acid, ethylene, and jasmonic acid (Zhang et al., 2024b). A total of 28 genes were identified in Auxin signaling, including 4 associated with auxin transporter 1 protein (AUX1), 1 linked to transport inhibitor response 1 protein (TIR1), 9 related to the auxin-responsive protein IAA (AUX/IAA), 2 corresponding to auxin response factors (ARFs) that function as transcription factors regulating the expression of growth hormone response genes, 1 from the auxin-responsive Gretchen Hagen 3 (GH3) gene family, and 11 belonging to the small auxin upregulated RNA (SAUR) family of proteins. Compared with those in the CK treatment, some of these genes were upregulated in the N1 or N2 treatment groups. For example, the DN6107_c0_g1 gene, identified as an ARF, exhibited fold changes of 5.71 and 4.89 in N1 and N2, respectively. In contrast, its expression level in CK was significantly lower, at only 1.98. In addition, DN13125_c0_g1 (AUX1), DN31919_c0_g2 (GH3), DN58737_c0_g1 (SAUR), DN3307_c0_g1 (SAUR) and DN17293_c0_g1 (SAUR) were expressed at higher levels in N1 and N2 than in CK. The results pertaining to auxin signal transduction demonstrated that the application of N resulted in the upregulation of the expression of AUX/IAA, ARF, SAUR, and GH3 genes.

### 3.6 DEGs involved in nitrogen metabolism and carbohydrate metabolism

**Figure 5:**
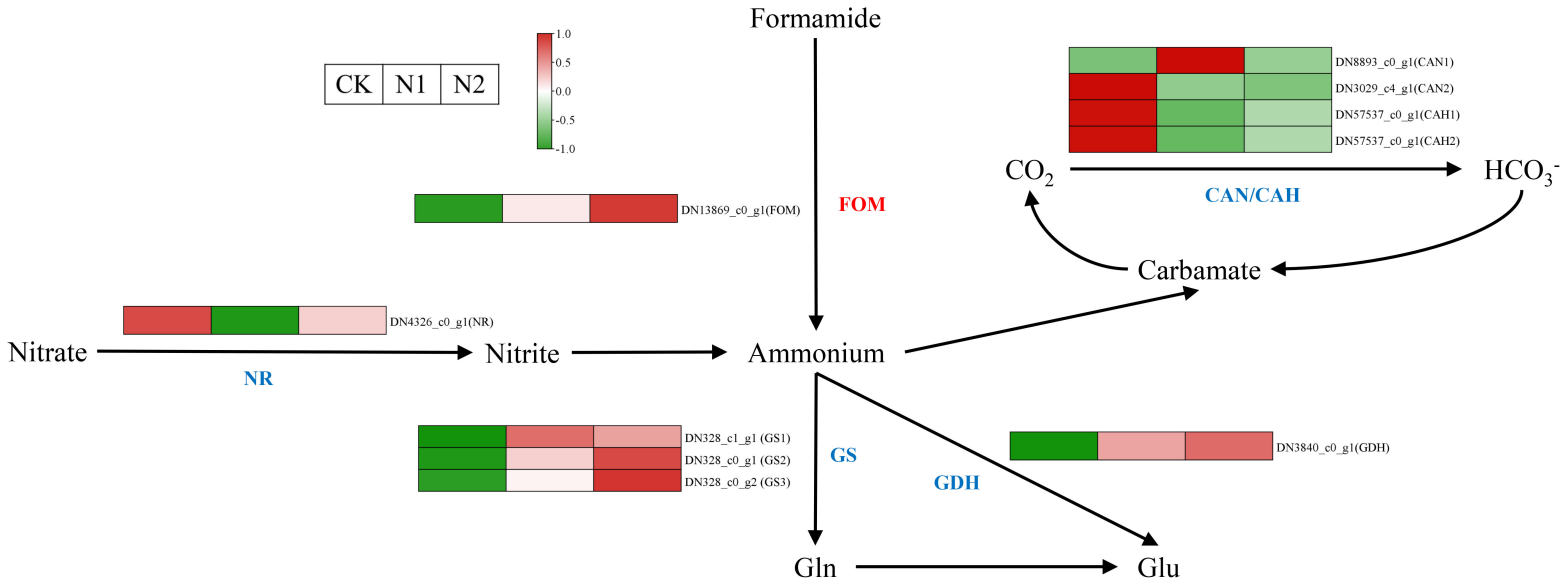
N metabolism pathway in *B balsamifera* leaves grown under different N regimes. The known enzyme names are shown in blue, and the unknown names are shown in red. FOM, Formamidase-like; CAN/CAH, Carbonic anhydrase. Each column represents a different N treatment (from left to right: CK, N1 and N2), as shown in the upper left corner. The average gene intensity is a key color scale according to the scale in the upper left corner. Red represents upregulation, whereas green represents downregulation.

N metabolism is very important for plant growth and development (Tang et al., 2019), and N metabolism in *B. balsamifera* leaves is strongly influenced by different N treatments. Nitrate reductase (NR), glutamine synthetase (GS) and glutamate dehydrogenase (GDH) are the key enzymes involved in N metabolism, and the expression of these genes encoding those enzymes is notably affected by different N regimens (**Figure 5**). Interestingly, the gene (DN4326_c0_g1) encoding NR was more highly expressed in the CK treatment than in the N1 and N2 treatments, which might be related to the stress response of *B. balsamifera* to adapt to the N deficiency environment. Three genes (DN328_c1_g1, DN328_c0_g1 and DN328_c0_g2) encoding GS and one gene (DN3840_c0_g1) encoding GDH were more highly expressed in the N1 and N2 treatments than in the CK. This suggested that more inorganic N was assimilated into organic N in the N1 and N2 treatments than in the CK, which led to higher N contents in the N1 and N2 treatments than in the CK in October (**Figure 1H**).

Carbohydrate metabolism is tightly linked to N metabolism (Saiz-Fernández et al., 2017). To further investigate the regulatory effects of different N supply levels on carbohydrate metabolism in *B. balsamifera* leaves at the transcriptional level, we screened DEGs related to carbohydrate metabolism. As shown in **Supplementary Figure S5**, most of the DEGs were downregulated in the N1 and N2 treatment groups compared with those in the CK group. Among these DEGs related to carbohydrate metabolism, 37 genes (6 upregulated and 31 downregulated) were involved in starch and sucrose metabolism (Ko00500) in the N1 vs. CK comparison (**Supplementary Figure S5A**), 30 genes (8 upregulated and 22 downregulated) were involved in amino sugar and nucleotide sugar metabolism (Ko00520) in the N1 vs. CK comparison (**Supplementary Figure S5B**), 21 genes (7 upregulated and 14 downregulated) were involved in starch and sucrose metabolism (Ko00500) in the N2 vs. CK comparison (**Supplementary Figure S5C**), 11 genes (1 upregulated and 10 downregulated) were involved in ascorbate and aldarate metabolism (Ko00053) in the N2 vs. CK comparison (**Supplementary Figure S5D**), and 3 downregulated genes were involved in amino sugar and nucleotide sugar metabolism (Ko00520) in the N2 vs. N1 comparison (**Supplementary Figure S5E**). These results indicated that N application promoted N metabolism and inhibited carbohydrate metabolism.

### 3.7 DEGs involved in terpenoid backbone biosynthesis, monoterpenoid biosynthesis and flavonoid biosynthesis

**Figure 6:**
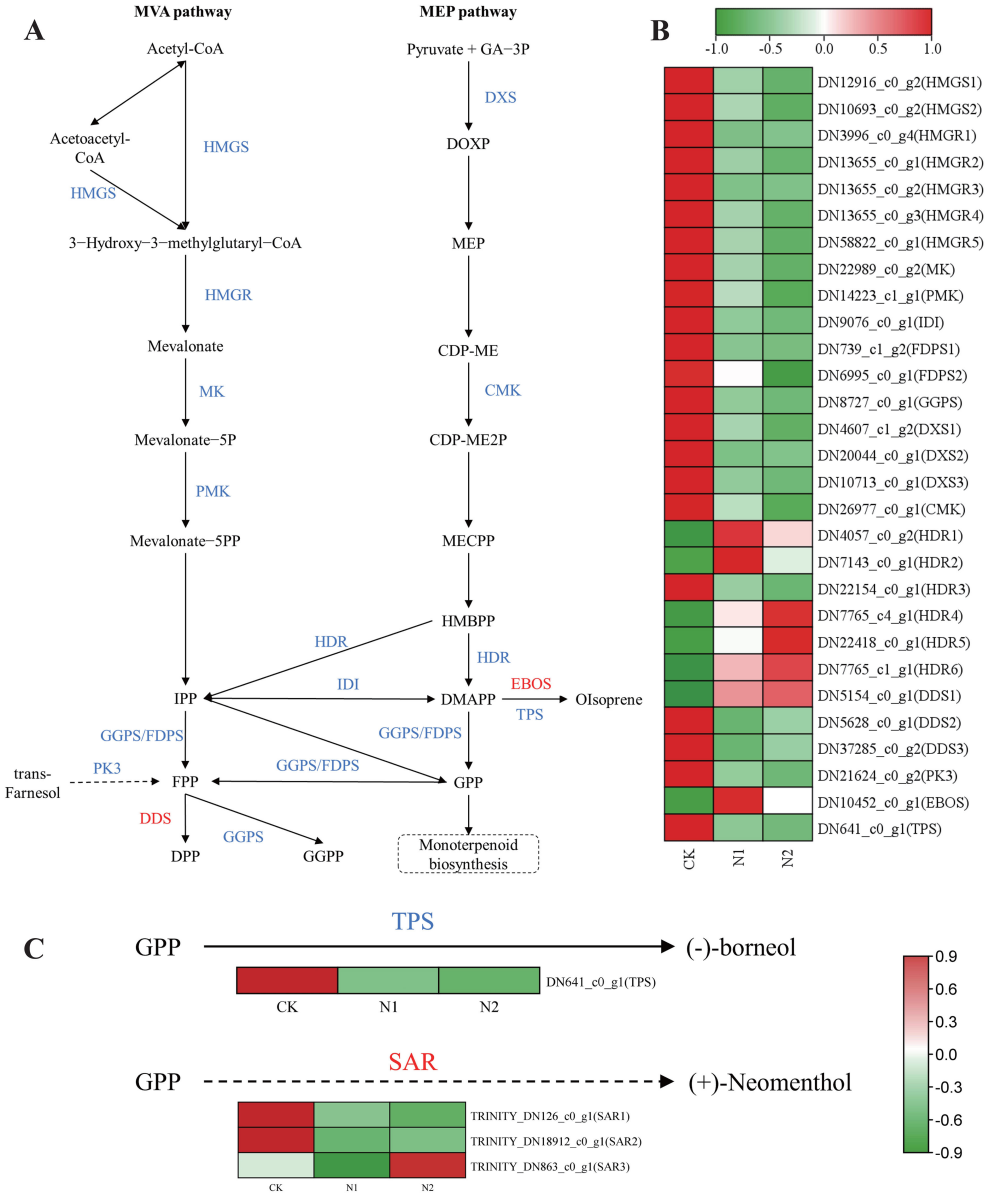
Biosynthesis pathway of diterpene lactones/monoterpenoids and heatmap analysis of their DEGs. **(A)** Predicting the biosynthetic pathway of diterpene lactones. **(B)** Heatmap of DEGs in the diterpene lactone pathway. **(C)** Prediction of the biosynthetic pathway of monoterpenoids and heatmap of monoterpenoid pathway DEGs. The solid arrows represent known steps, and the unknown steps are indicated by dashed arrows. The known enzyme names are shown in blue, and the unknown names are shown in red. HMGS, 3-hydroxy-3-methylglutaryl-1 CoA synthase; HMGR, 3-hydroxy-3-methylglutaryl CoA reductase; MK, mevalonate kinase; PMK, phosphomevalonate kinase; IDI, Ispentenyl diphosphate isomerase; FDPS, farnesyl diphosphate synthase; GPPS, geranylgeranyl diphosphate synthase; DXS, 1-deoxy-D-xylulose-5-phosphate synthase; CMK, 4-diphosphocytidyl-2-C-methyl-D-erythritol kinase; HDR, 4-hydroxy-3-methylbut-2-enyl diphosphate reductase; PK3, phytol kinase 3; TPS, terpenoid synthase.

(-)-Borneol, a bicyclic monoterpene, is synthesized through the terpenoid backbone biosynthesis pathway (Ko00900) (**Figure 6A**) and the monoterpenoid biosynthesis pathway (Ko00902) (**Figure 6C**) (Guan et al., 2016). Recent studies (Yu et al., 2024) have shown that DMAPP and IPP, the key precursors required for terpene synthesis, are produced via the MVA and MEP pathways (**Figure 6A**). The (-)-borneol synthesis pathway in chloroplasts is relatively short, with only one step in which terpenoid synthase (TPS) converts geranyl diphosphate (GPP) into (-)-borneol (**Figure 6C**) (Ma et al., 2022; Chen et al., 2023c). According to the transcriptomic analysis results, 29 DEGs were annotated to terpenoid backbone biosynthetic pathways (**Figure 6B**). A heatmap was drawn on the basis of the TPM values of the DEGs in *B. balsamifera* leaves, which revealed differences in the expression of diterpene lactone biosynthesis-related genes among the CK, N1 and N2 treatments. The highest expression levels were detected in *B. balsamifera* leaves in the CK treatment, followed by those in the N1 and N2 treatments. This finding is consistent with our results, which revealed that (-)-borneol was most abundant in the leaves of the plants in the CK treatment group (**Figure 2A**). In the MEP pathway, we annotated 3DXS, 1CMK, and 6HDR genes. Among these genes, DXS (DN4607_c1_g2 and DN10713_c0_g1), CMK (DN26977_c0_g1), and HDR (DN22154_c0_g1) presented greater expression in the CK treatment than in the N1 and N2 treatments, with TPM values of 66.33, 50.31, 21.31, and 64.36, respectively. These enzymes may represent key nodes in monoterpene and diterpene biosynthesis. The TPM values of the TPS (DN641_c0_g1) gene in the CK, N1 and N2 treatments were 91.33, 24.71 and 18.48, respectively (**Figure 6C**).

The flavonoid metabolic pathways in *B. balsamifera* leaves treated with different N regimens are shown in **Supplementary Figure S6**. Although numerous flavonoids have been isolated from *B. balsamifera*, the expression of genes involved in flavonoid biosynthesis in this species in response to different N levels has not been reported. Here, we mined the *B. balsamifera* transcriptome and identified 12 DEGs in the N1 vs. CK comparison (**Supplementary Figure S6**, **Figure 3A**) and 10 DEGs in the N2 vs. CK comparison (**Supplementary Figure S6**, **Figure 3B**). Most of the DEGs were highly expressed in the CK treatment, which is consistent with our total flavonoid content results (**Figure 2B**). Among these genes, CHS2 (DN6376_c0_g1), CHS3 (DN48530_c0_g1), CHI1 (DN8666_c0_g2), CHI2 (DN3898_c0_g1), F3H (DN5329_c1_g1), TCM4 (DN1783_c0_g3), C3’H2 (DN4041_c0_g1), HCT (DN60667_c0_g1), FLS (DN18840_c0_g3), CCOM1 (DN7647_c0_g2) and CCOM2 (DN16775_c0_g1) were significantly upregulated in the CK treatment, suggesting that these enzyme activities may be inhibited by N application.

### 3.8 Analysis of TFs in different nitrogen regimes

**Figure 7:**
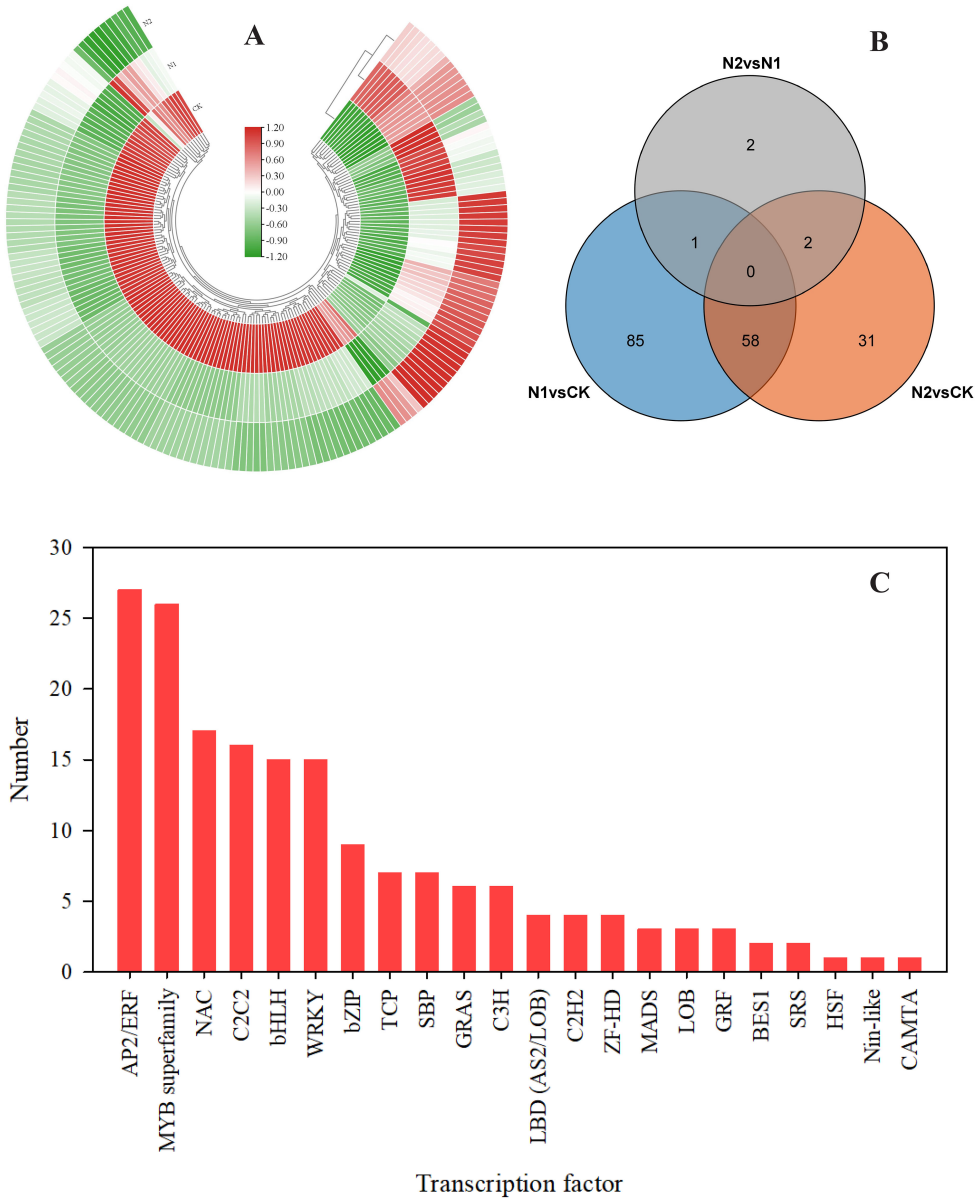
Overview of the transcription factors identified from the DEGs. **(A)** Heatmap showing 179 DEGs identified as transcription factors (TFs). **(B)** Venn diagram illustrating overlaps among different TFs in different comparison groups, i.e., the N1 vs. CK, N2 vs. CK, and N2 vs. N1 comparisons. **(C)** A total of 179 DEGs were divided into 22 transcription factor families.

Transcription factors (TFs) play crucial roles in either promoting or suppressing the expression of genes involved in biosynthetic pathways, thus influencing the production and buildup of secondary metabolites in plants (Yang et al., 2012; Cao et al., 2020). To understand the changes in TFs under different N treatments, we used iTAK 1.2 software to analyze *B. balsamifera* TFs (Zheng et al., 2016). As shown in **Figure 7A**, a total of 179 unigenes were identified as TFs. A total of 144 differentially expressed TFs were identified in the N1 vs. CK comparison. A total of 91 TFs were identified in the N2 vs. CK comparison. Only 5 TFs were identified in the N2 vs. N1 comparison (**Figure 7B**). Among these TFs, the top six most enriched TF families in the different N treatments were AP2/ERF (27), MYB (26), NAC (17), C2C2 (16), bHLH (15), and WRKY (15) (**Figure 7C**).

### 3.9 Validation of genes via quantitative real-time PCR

**Figure 8:**
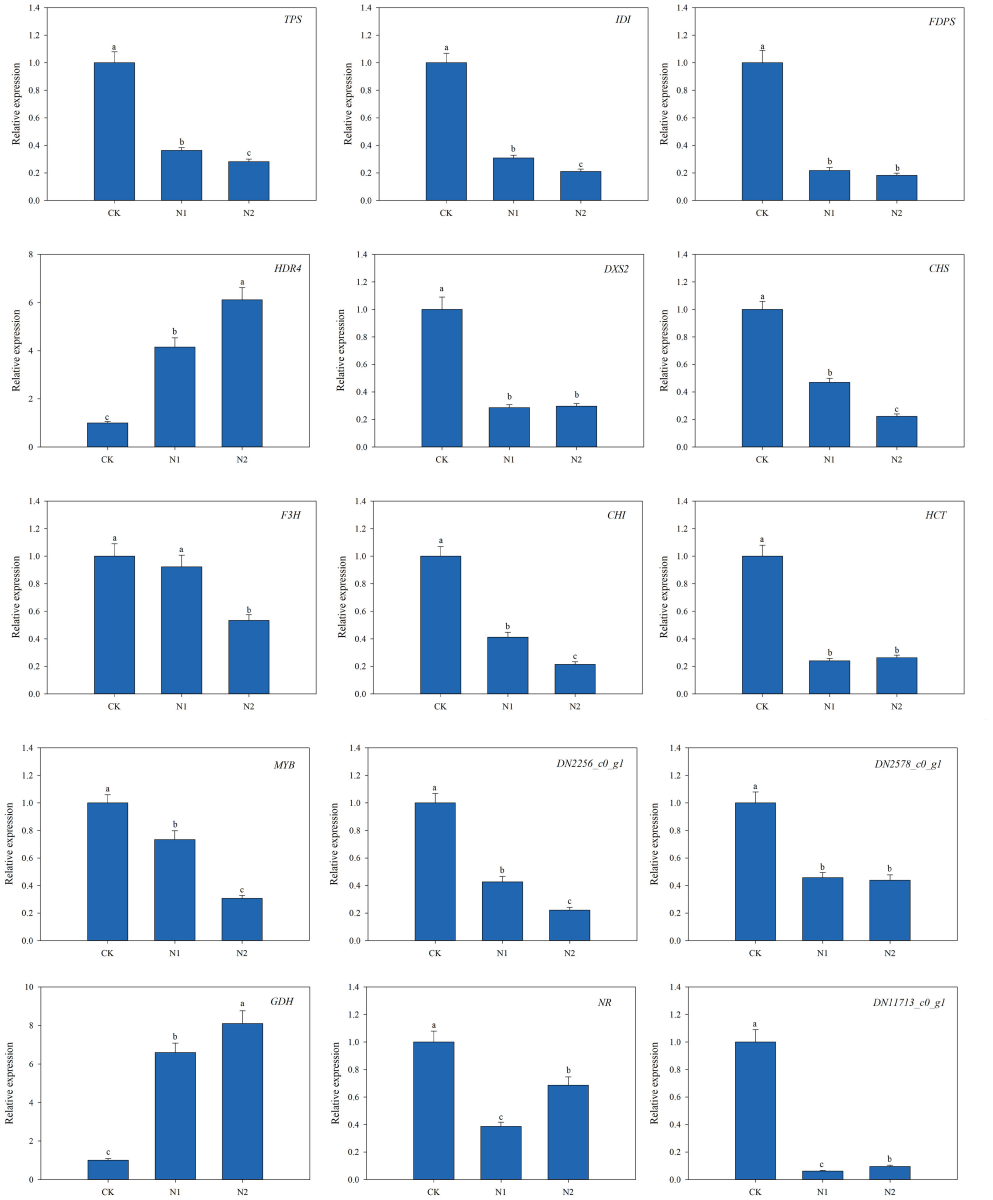
Quantitative real-time PCR (qRT−PCR) validation of 15 differentially expressed genes (DEGs). Different letters at the top of the histogram bars denote significant differences among treatments (*p*<0.05). The vertical bars indicate the standard deviation (SD) (n = 3).

To confirm the reliability of the RNA-Seq results, 15 representative genes were chosen on the basis of the above analysis, and their expression levels in the leaves of *B. balsamifera* plants treated with different N regimens were validated via quantitative real-time PCR (qRT−PCR) (**Figure 8**). The 15 representative genes encoded *TPS*, *IDI*, *FDPS*, *HDR4*, *DXS2*, *CHS*, *F3H*, *CHI*, *HCT*, *MYB* (TF), *GDH*, *NR*, NADH-cytochrome b5 reductase (*DN2256_c0_g1*), endoglucanase 17 (*DN11713_c0_g1*) and fructose-1,6-bisphosphatase (*DN2578_c0_g1*), which are key proteins or enzymes involved in monoterpenoid biosynthesis, terpenoid backbone biosynthesis, flavonoid biosynthesis, N metabolism, starch and sucrose metabolism and the pentose phosphate pathway. Interestingly, our analysis revealed that most of the tested genes were significantly upregulated in the CK treatment, whereas the expression levels of *HDR4* and *GDH* were upregulated in the N1 and N2 treatments. Moreover, the comparative analysis of the expression patterns of these genes from the RNA-Seq and qRT−PCR results revealed a fairly good match, which indicated the reliability of the RNA-Seq results used in this study.

## 4 Discussion

*B. balsamifera* is an economical crop with significant potential in the medical and industrial sectors (Guan et al., 2024a). N has a strong effect on the growth and accumulation of medicinal components in medicinal plants. However, few studies have investigated the effects of different N application rates on growth, (-)-bornol synthesis and leaf gene expression in *B. balsamifera*.

### 4.1 *B. balsamifera* growth was promoted by moderate nitrogen application

In this study, we found that *B. balsamifera* growth was strongly promoted by N application, and compared with those under the CK treatment, the leaf numbers, leaf SPAD values and leaf dry weights of *B. balsamifera* under the N1 and N2 treatments were greatly elevated (**Figure 1**). N is a component of proteins and nucleic acids and is also the basic component of many coenzymes, cogroups (such as NAD, NADP, and FAD), chlorophyll molecules and some plant hormones (such as auxin and cytokinin) and vitamins (such as VB1, VB2, VB6, and PP). The contribution of N to crop yield can reach 40%~50% (Sylvester-Bradley and Kindred, 2009; Chu et al., 2021). For medical cannabis (*Cannabis sativa* L.), the biomass of leaves consistently increased throughout the N range tested (30 mg/L ~ 320 mg/L). Under hydroponic conditions and field plot experiments, N application significantly promoted an increase in biomass in stevia (Sun et al., 2019). With increasing N application rates (0 kg N ha^-1^ ~ 250 kg N ha^-1^), the biomass of *B. balsamifera* increased to the highest value (Lan et al., 2017). Therefore, these results are consistent with the conclusions of this study. Additionally, three genes encoding GS, specifically DN328_c1_g1, DN328_c0_g1 and DN328_c0_g2, along with one gene encoding GDH, DN3840_c0_g1, exhibited significantly higher expression levels in the N1 and N2 treatments compared to the CK (**Figure 5**). Ammonium (NH_4_^+^) are converted into glutamine by the enzyme GS, a process essential for the subsequent reaction involving glutamate synthetase (GOGAT) that leads to the production of glutamate. This pathway is referred to as the GS/GOGAT cycle. In addition, NH_4_^+^ can also be converted to glutamine through the action of GDH, which serves as an alternative mechanism to improve ammonium tolerance (Song et al., 2022). Based on the analysis of gene expression heatmaps from paper mulberry transcriptomes, the expression levels of GS showed a significant increase following moderate N application, and moderate N also enhanced the growth and biomass production of paper mulberry (Ni et al., 2020). Furthermore, in the context of low N treatment, the height and fresh weight of transgenic rice plants overexpressing ScAMT1.1 were found to be 36.48% and 51.55% greater, respectively, than those of the wild type. Concurrently, the activity of key enzymes associated with ammonium assimilation, including GS and GDH, as well as the expression levels of essential ammonium assimilation genes, specifically GS, GDH and GOGAT, were significantly increased in the transgenic plants relative to the wild type (Gao et al., 2023).

**Figure 9:**
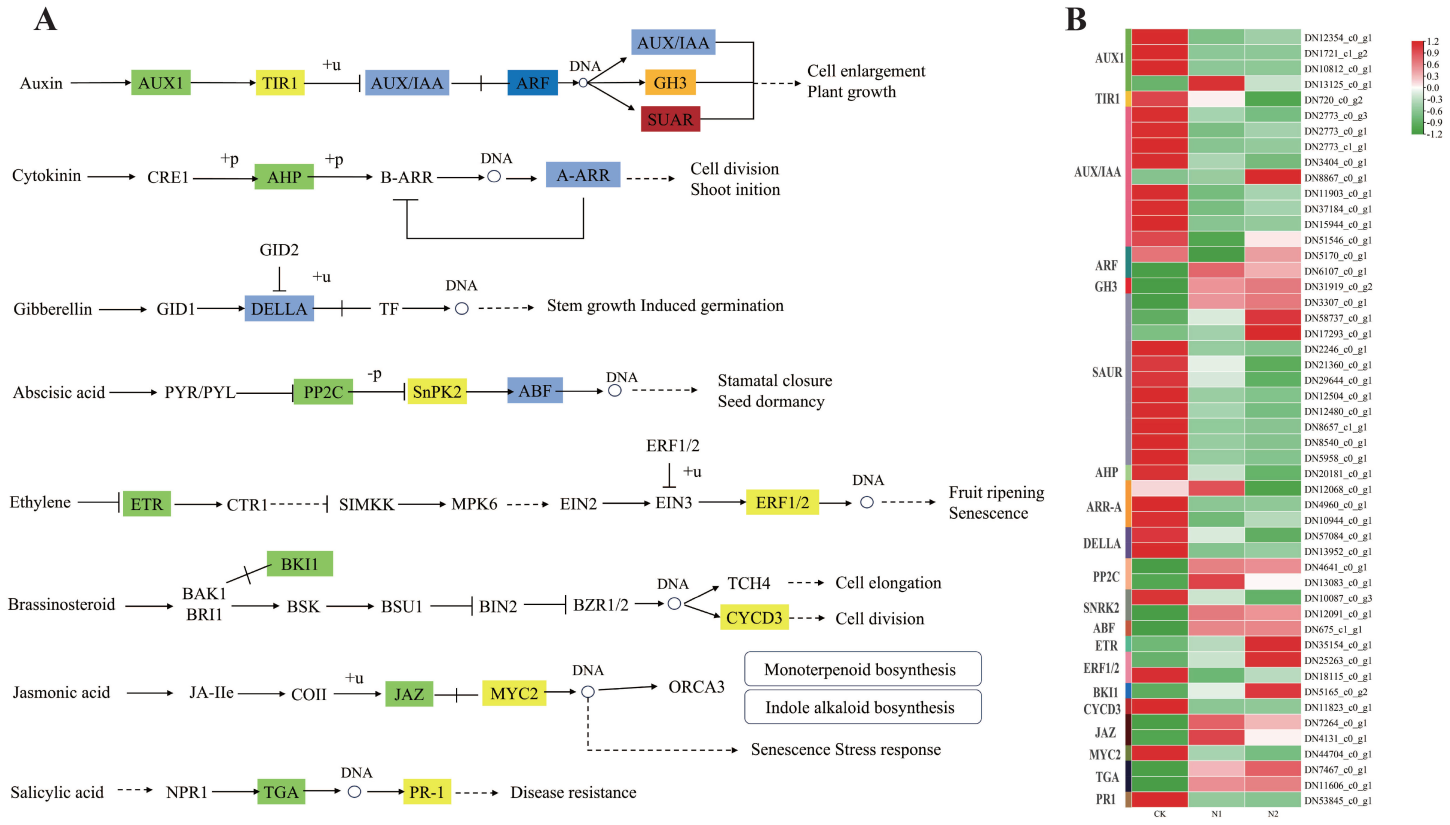
KEGG pathway analysis revealed significant enrichment in plant hormone signal transduction. **(A)** A schematic representation was provided to illustrate the plant hormone signal transduction pathways associated with the DEGs identified in this study. **(B)** Key enzymes were depicted as colored shaded rectangles, with the corresponding genes and their expression levels presented in the accompanying heatmap.

Auxin promotes plant growth, and auxin synthesis is related to N and auxin-related gene expression (Li et al., 2020). In this study, the genes associated with auxin synthesis, namely, DN6107_c0_g1 (ARF), DN13125_c0_g1 (AUX1), DN31919_c0_g2 (GH3), DN58737_c0_g1 (SAUR), DN3307_c0_g1 (SAUR), and DN17293_c0_g1 (SAUR), exhibited significantly higher expression levels in the N1 and N2 treatments compared to the CK (**Figure 9**). The 15 SAUR genes and major transcription factors (auxin/indole-3-acetic acid (AUX/IAA)) in flag leaves of wheat were expressed at higher levels under 210 kg N ha^-1^ than under 0 kg N ha^-1^ (Effah et al., 2022). In addition, N application could significantly increase the auxin content in tea leaves, and the auxin content in tea leaves increased with increasing N application (Yuan et al., 2016a). N is a major element in the synthesis of proteins, which are precursors of the growth hormone indoleacetic acid (IAA) (Chu et al., 2021). Therefore, the application of moderate N might promote protein synthesis and then provide raw materials for auxin synthesis in *B. balsamifera* leaves. However, with a further increase in the N application rate, there was no significant difference in the growth indices between the N1 and N2 treatments (**Figure 1**). Numerous plants exhibit toxicity symptoms and reduced growth when exposed to an overabundance of nutrients, especially N (Saloner and Bernstein, 2021; Yan et al., 2024; Zhang et al., 2024a). For example, elevated N levels result in an imbalanced distribution of energy within plants, which is evident in excessive leaf growth and the suppression of root development (Chen et al., 2016). Moreover, an overabundance of N can increase the susceptibility of plants to environmental stressors and diseases (Wang et al., 2023). Additionally, elevated N concentrations in the soil can lead to an increased rate of root respiration, which may subsequently inhibit root viability and diminish water and N absorption by plants (Yan et al., 2024). The accumulation of hydrogen peroxide (H_2_O_2_) induced by high N stress led to elevated levels of malondialdehyde (MDA) in the roots of giant reed plants. Furthermore, the root activity of giant reed plants subjected to high N stress was significantly lower than that of plants in the moderate N group (Zhang et al., 2024a). Therefore, further study is needed to determine the effects of N application on the physiological characteristics of the *B. balsamifera* root system.

### 4.2 (-)-Borneol synthesis in *B. balsamifera* leaves was inhibited by excessive nitrogen

The primary objective of this study was to increase the yield of (-)-borneol in *B. balsamifera* cultivation. This study revealed that moderate N application significantly increased the yield of (-)-borneol, whereas excessive N application led to a substantial decrease in the content of (-)-borneol in leaves (**Figure 2A**). In this study, the leaf N content and photosynthetic capacity (leaf number and SPAD value) of *B. balsamifera* plants significantly decreased in the CK treatment, resulting in reduced *B. balsamifera* biomass (**Figure 1**). However, the contents of (-)-borneol and total flavonoids, important functional products in *B. balsamifera* leaves, were significantly greater in the CK treatment in October (**Figure 2**). From an agronomic standpoint, this outcome was linked to the ‘concentration effect’ resulting from the decreased leaf biomass in the CK treatment (**Figure 1**). On the other hand, due to competition for carbon and energy, there is a trade-off between primary metabolism (plant growth) and secondary metabolism (accumulation of medicinal ingredients) (Sun et al., 2021). This growth−differentiation balance also implies that plant growth inhibition occurs through an improvement in secondary metabolism, including in the biosynthesis of terpenoids and flavonoids (Sun et al., 2019). Similar phenomena have also been documented in *Centella asiatica* (Muller et al., 2013), *Cannabis sativa* L. (Saloner and Bernstein, 2021) and Tibetan hulless barley (Wei et al., 2016). These findings indicate that the trade-off between *B. balsamifera* leaf biomass and (-)-borneol content/total flavonoid content is a result rather than a cause of plant metabolic responses to N availability, and it is crucial to delve deeper into the mechanisms underlying these phenomena.

Several previous reports have demonstrated that the biosynthesis of terpenoids or flavonoids increased in N-deficient *Stevia rebaudiana* (Sun et al., 2021), *Panax notoginseng* (Cun et al., 2024) and *Robinia pseudoacacia* (Li et al., 2024), which increased their tolerance to N deficiency. These findings are consistent with our results showing significantly increased (-)-borneol and total flavonoid contents in *B. balsamifera* leaves in the CK treatment (**Figure 2**). In addition, increased expression levels of most genes (*HMGS*, *HMGR*, *MK*, *PMK*, *IDI*, *FDPS*, *GGPS*, *DXS*, *CMK*, *TPS*, *CHI*, *F3H*, *HCT*, and *FLS*), which are involved in terpenoid backbone biosynthesis, monoterpenoid biosynthesis and flavonoid biosynthesis, respectively, were detected (**Figure 6**; **Supplementary Figure S6**). These findings suggest a notable increase in terpenoid and flavonoid synthesis in plants subjected to N deficiency. Notably, these results further validate our hypothesis that low N availability promotes the accumulation of C-containing secondary metabolites, such as terpenoids and flavonoids, which do not contain any N atoms (Saloner and Bernstein, 2021). Sugars are crucial components in the synthesis of terpenoids or flavonoids (Yang et al., 2018). N deficiency triggered a rise in *Stevia rebaudiana* leaf sugar levels and increased the activity of genes encoding enzymes within the MEP pathway or terpene production module, thereby fostering terpenoid accumulation (Sun et al., 2021). Interestingly, in the present study, KEGG enrichment analysis revealed that N deficiency stimulated the expression of genes related to carbohydrate metabolism, including “starch and sucrose metabolism”, “amino sugar and nucleotide sugar metabolism”, and “ascorbate and aldarate metabolism” (**Supplementary Figures S5**), and secondary metabolism, including “terpenoid backbone biosynthesis” and “monoterpenoid biosynthesis” (**Figure 6**). As anticipated, the increased (-)-borneol levels could be linked to increased gene expression related to carbohydrate metabolism, terpenoid backbone biosynthesis and monoterpenoid biosynthesis in *B. balsamifera* plants grown under conditions of N deficiency.

### 4.3 MYB transcription factors regulate (-)-borne biosynthesis in response to the nitrogen regime

Transcription factors (TFs) can either activate or suppress gene expression in biosynthetic pathways, thus controlling the synthesis and accumulation of plant secondary metabolites (Feng et al., 2020). A recent study revealed that TFs, AP2/ERF, bHLH, MYB, NAC, WRKY and bZIP, are involved in terpenoid synthesis (Dong et al., 2020). Similar to the findings of a recent study, our results revealed 179 DEGs in response to different N regimes, including AP2/ERF, MYB, NAC, C2C2, bHLH, and WRKY, which were the six most enriched TF families (**Figure 8C**). MYBs constitute one of the most expansive families of TFs in plants, orchestrating transcriptional control over terpenoid biosynthesis and various other secondary metabolites (Cao et al., 2020). In this study, a total of 26 MYB TFs were identified (**Figure 7C**), 19 of which were upregulated in response to CK treatment. In foxtail millet, a total of 25 MYB TFs were identified in response to low-nitrogen stress. Among these, 20 TFs were found to be upregulated, while 5 TFs exhibited downregulation (Ge et al., 2019). In our previous study, 32 *1R-BbMYB* and 15 *R2R3-BbMYB* genes were identified in *B. balsamifera* (Guan et al., 2020). Numerous studies have demonstrated the positive role of MYB TFs in the synthesis of terpenoid compounds, such as (E)-beta-caryophyllene in *Dendrobium officinale* (Lv et al., 2022), with anolide in *Withania somnifera* (Singh et al., 2024) and cannabinoids in *Cannabis sativa* (Tang et al., 2023). Additionally, in *Panax notoginseng*, 10 MYB TFs are significantly induced under N stress (Chen et al., 2023b). Furthermore, the coordinated enhancement of MYB TF gene expression and terpenoid/flavonoid accumulation under N deficiency conditions has been documented (Nemie-Feyissa et al., 2014; Sun et al., 2021). These results, together with our findings, suggest an N-MYB-terpenoid module of action in N deficiency-stimulated (-)-borneol synthesis. In light of the findings of previous studies and our study, we propose that MYB TFs regulate (-)-borneol biosynthesis in response to the N regime, but the specific underlying mechanisms need to be explored in the future.

## 5 Conclusions

Our study demonstrated that N deficiency (CK, control) significantly restrained the growth of *B. balsamifera*. In contrast, biomass and (-)-borneol accumulation were found to be positively correlated, with the highest yield of (-)-borneol achieved at a N application rate of 150 kg N ha^-1^ (N1 treatment). Notably, the application of 300 kg N ha^-1^ (N2 treatment) did not increase plant growth and markedly inhibited (-)-borneol synthesis in *B. balsamifera* compared with the N1 treatment. To elucidate the molecular mechanisms underlying the different leaf responses to the CK, N1, and N2 treatments, we conducted an RNA-Seq analysis of leaf samples collected after exposure to these various N regimes to investigate gene expression patterns. Our in-depth analysis revealed that the expression of several genes associated with auxin signaling and N metabolism was stimulated by N application. Conversely, most genes involved in carbohydrate metabolism and secondary metabolism, including those related to terpenoid backbone biosynthesis, monoterpenoid biosynthesis, and flavonoid biosynthesis, were upregulated under N deficiency. Furthermore, the terpenoid synthase gene (*BbTPS*) and *MYB* transcription factors (TFs) appear to be key regulators of (-)-borneol synthesis driven by N deficiency. This research provides valuable insights into the synthesis of (-)-borneol and total flavonoids in response to N availability, with important implications for the future cultivation of *B. balsamifera*.

## Data Availability

The original contributions presented in the study are publicly available. This data can be found at the National Center for Biotechnology Information (NCBI) using accession number PRJNA1099649, as well as in the article/**Supplementary Material**.
