# Supplementary material for: Integrated physiological and transcriptomic analysis uncovers the mechanism of moderate nitrogen application on promoting the growth and (-)-borneol accumulation of Blumea balsamifera
Source: Front Plant Sci. 2025 Jan 31;15:1531932. doi: 10.3389/fpls.2024.1531932 (PMC11825785; doi:10.3389/fpls.2024.1531932)
Supplement: Supplementary file 1 [file DataSheet1.zip › Supplementary files/Supplemental Figure and Table.docx]

**Integrated Physiological and Transcriptomic Analysis Uncovers the Mechanism of** **Moderate Nitrogen Application** **on Promoting the Growth and (-)-Borneol Accumulation** **of *Blumea balsamifera***

Yuan Yuan^1,†^, Wei-Jie Tang^2,3†^, Jia-Yuan Cao^2^, Ke Zhong^2,3^, Ze-Jun Mo^2^, Ying Zhou^2*^ and Yu-Xin Pang^2,4*^

1 School of Biosciences and Biopharmaceutics, Guangdong Pharmaceutical University, Guangzhou, 510006, China;

2 College of Pharmaceutical Sciences, Guizhou University of Traditional Chinese Medicine, Guiyang 550025, Guizhou, China;

3 Research Center for Ecological Planting Technology of Traditional Chinese and Ethnic Medicines, Guizhou University of Traditional Chinese Medicine, Guiyang 550025, Guizhou, China;

4 Yunfu Traditional Chinese Medicine Resources and Germplasm Resources Database Management Center, Yunfu, 527300, China;

* Correspondence: yingzhou71@sina.com (Ying Zhou); blumeachina@126.com (Yu-Xin Pang)

† These authors have contributed equally to this work.

**Supplemental Figures and Tables**

**Supplemental Figure S1.** Monthly mean temperature and total precipitation during the *B. balsamifera* growing season from June to December 2023.

**Supplemental Figure S2.** Principal component analysis (PCA) of the RNA-Seq output.

**Supplemental Figure S3.** GO enrichment analysis of all the DEGs in different N regimes. The top 20 enriched GO terms in the N1 vs. CK (**a**), N2 vs. CK (**b**), and N2 vs. N1 (**c**) comparisons are presented. The horizontal axis represents the rich factor, while the vertical axis represents the GO terms. Number: number of DEGs; Padjust: *p*-value < 0.05.

**Supplemental Figure S4.** KEGG enrichment analysis of all the DEGs in the N2 vs. N1 comparison. The horizontal axis denotes the enrichment factor, with the vertical axis indicating the pathway name. Number: number of DEGs; Padjust: *p* value < 0.01.

**Supplemental Figure S5.** Heatmap showing the expression profile of the DEGs involved in carbohydrate metabolism between different comparison groups. (a) Starch and sucrose metabolism in the N1 vs. CK comparison. (b) Amino sugar and nucleotide sugar metabolism in the N1 vs. CK comparison. (c) Starch and sucrose metabolism in the N2 vs. CK comparison. (d) Ascorbate and aldarate metabolism in the N2 vs. CK comparison. (e) Amino sugar and nucleotide sugar metabolism in the N2 vs. N1 comparison. Red represents upregulation, while green represents downregulation.

**Supplemental Figure S6.** Biosynthesis pathway of flavonoids and heatmap analysis of their DEGs. (a) Predicting the biosynthetic pathway of flavonoids. (b) Heatmap of DEGs in the biosynthetic pathway of flavonoids. The known enzyme names are shown in blue, and the unknown names are shown in red.

**Supplemental Table S1.** Information on the primers used for qRT‒PCR in this study. **Supplemental Table S2.** Summary statistics of sequence analysis.

**Supplemental Table S3.** Summary of the annotations of *B. balsamifera* compared with those in public databases.
