# Supplementary figures and images for: Integrated physiological and transcriptomic analysis uncovers the mechanism of moderate nitrogen application on promoting the growth and (-)-borneol accumulation of Blumea balsamifera
Source: Front Plant Sci. 2025 Jan 31;15:1531932. doi: 10.3389/fpls.2024.1531932 (PMC11825785; doi:10.3389/fpls.2024.1531932)

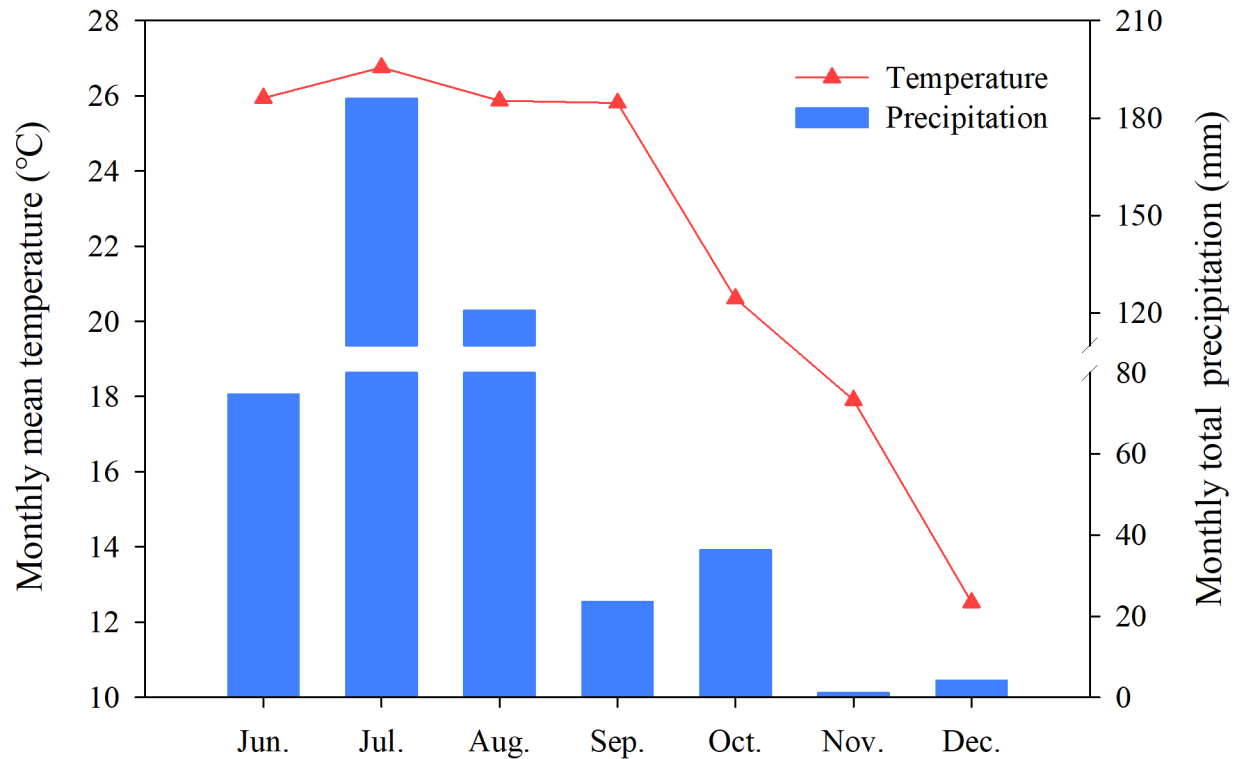

Supplement: Supplementary file 1 [file DataSheet1.zip › Supplementary files/Figure S1.pdf]

# PCA analysis

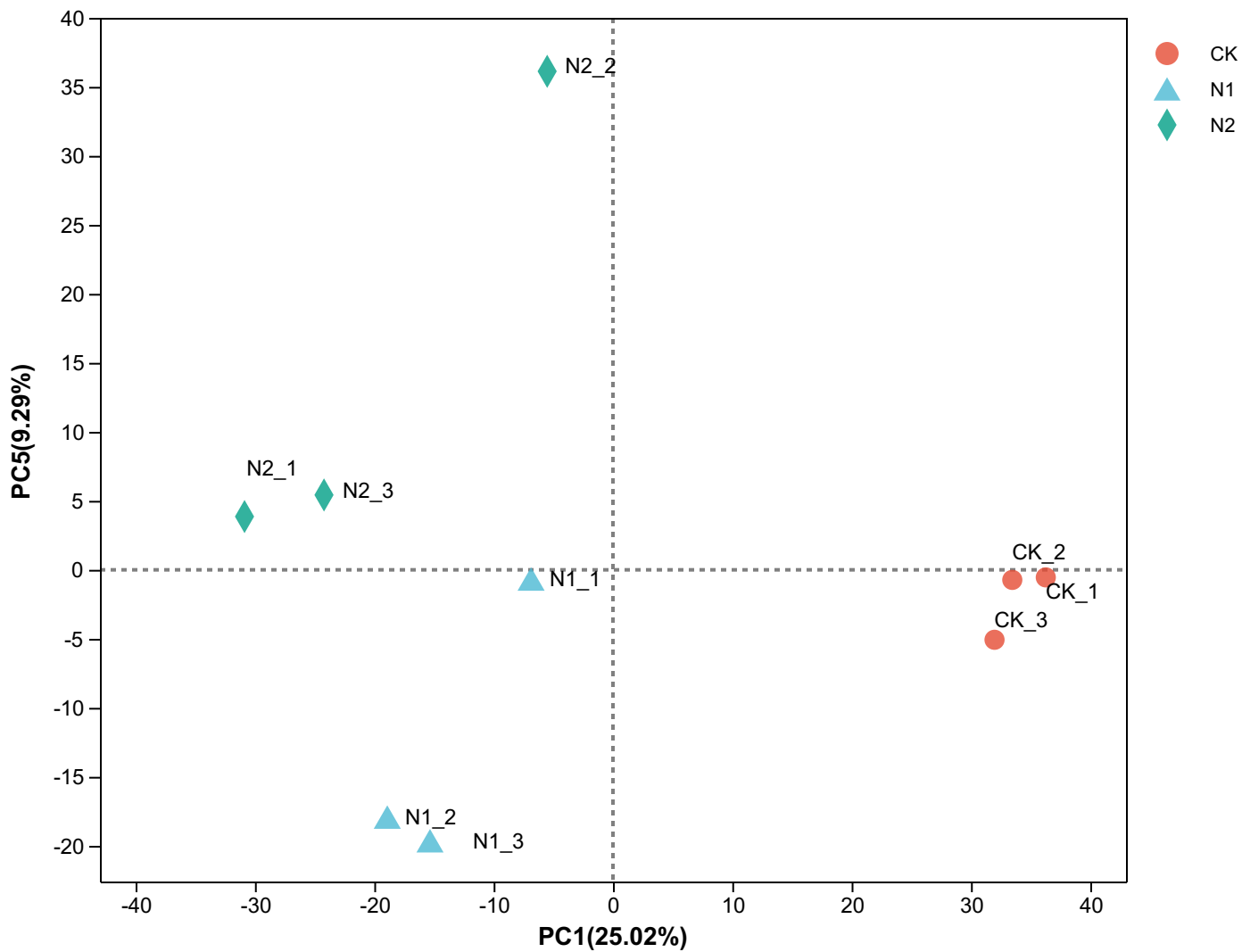

Supplement: Supplementary file 1 [file DataSheet1.zip › Supplementary files/Figure S2.pdf]

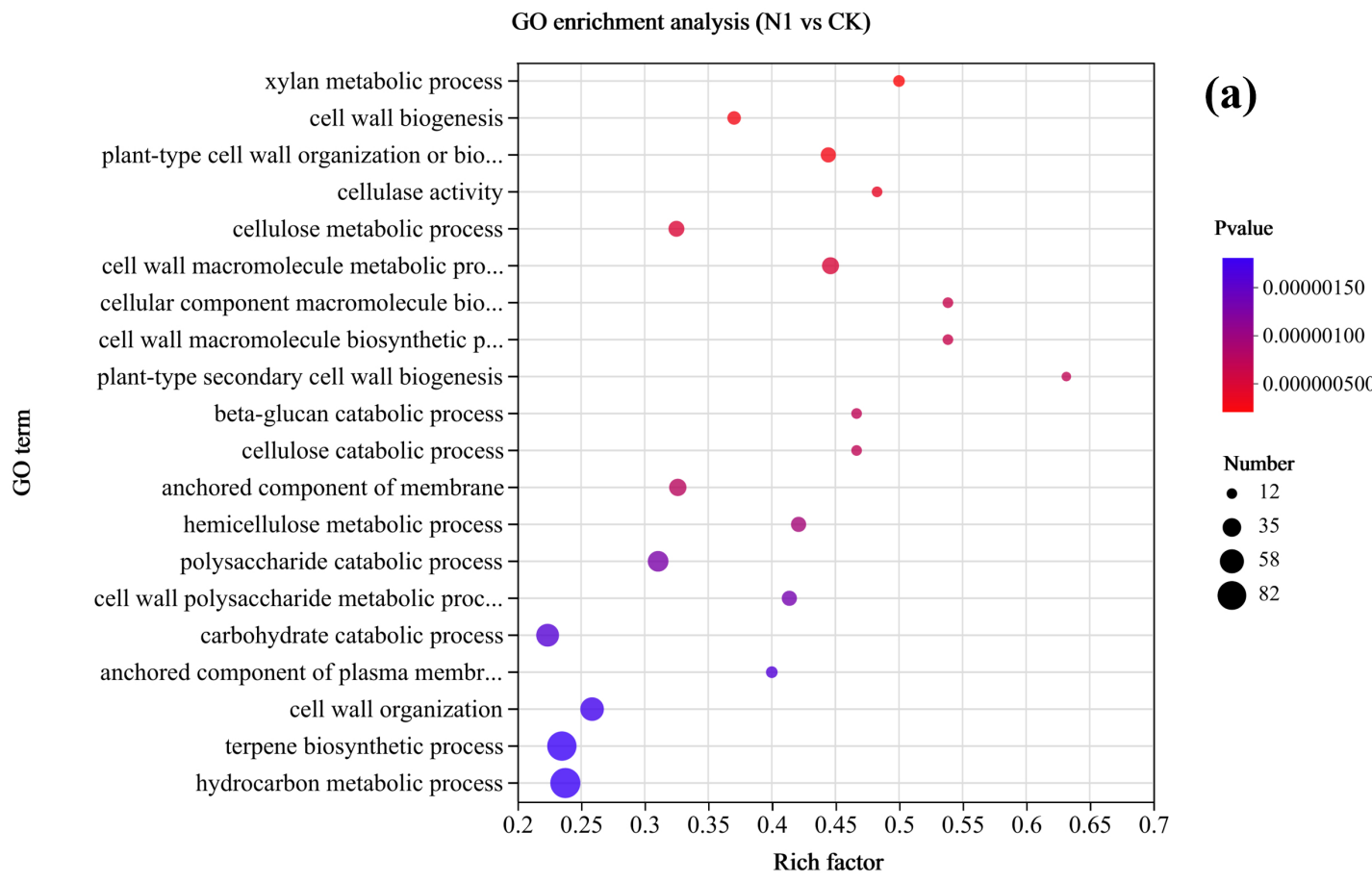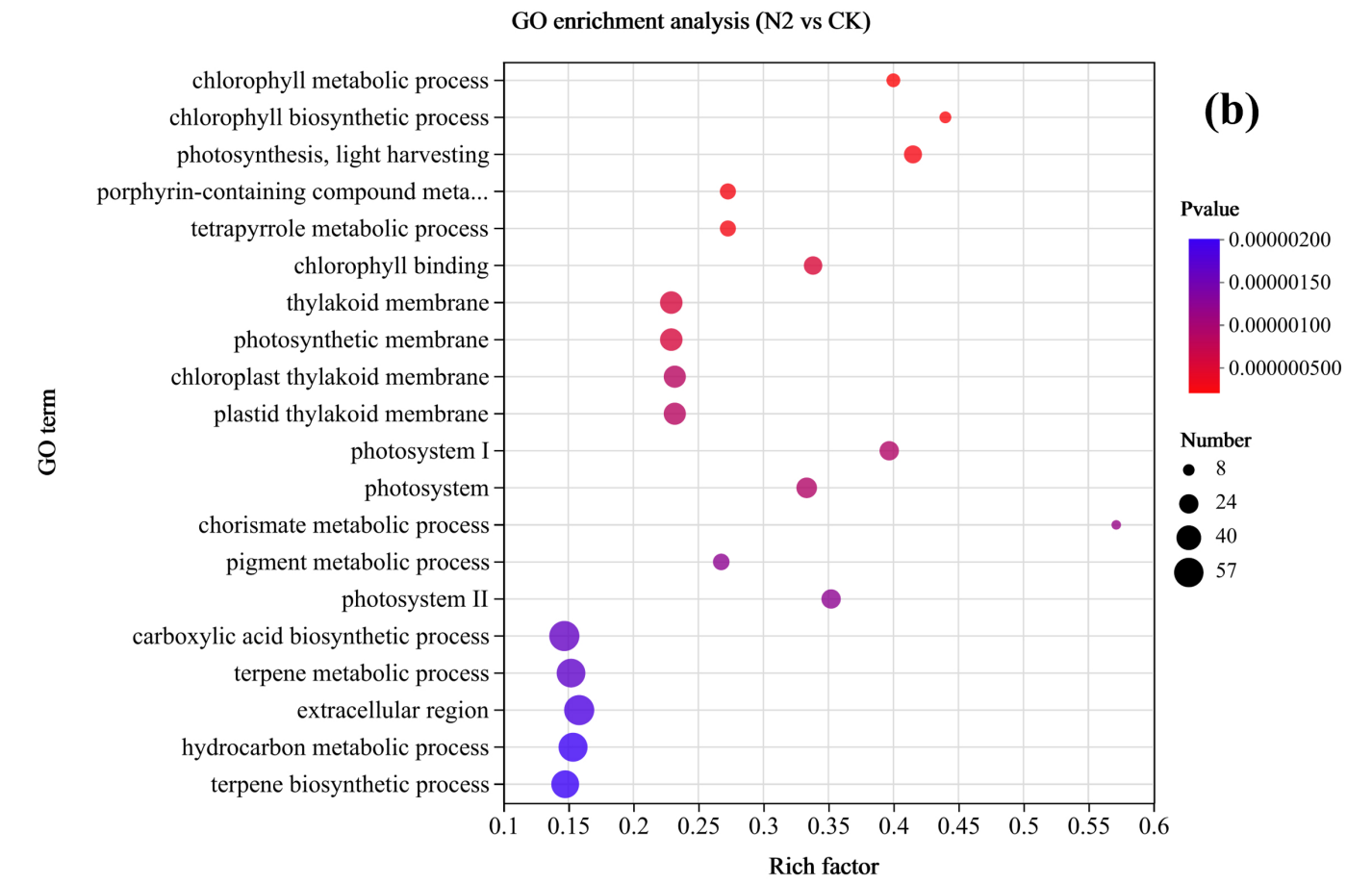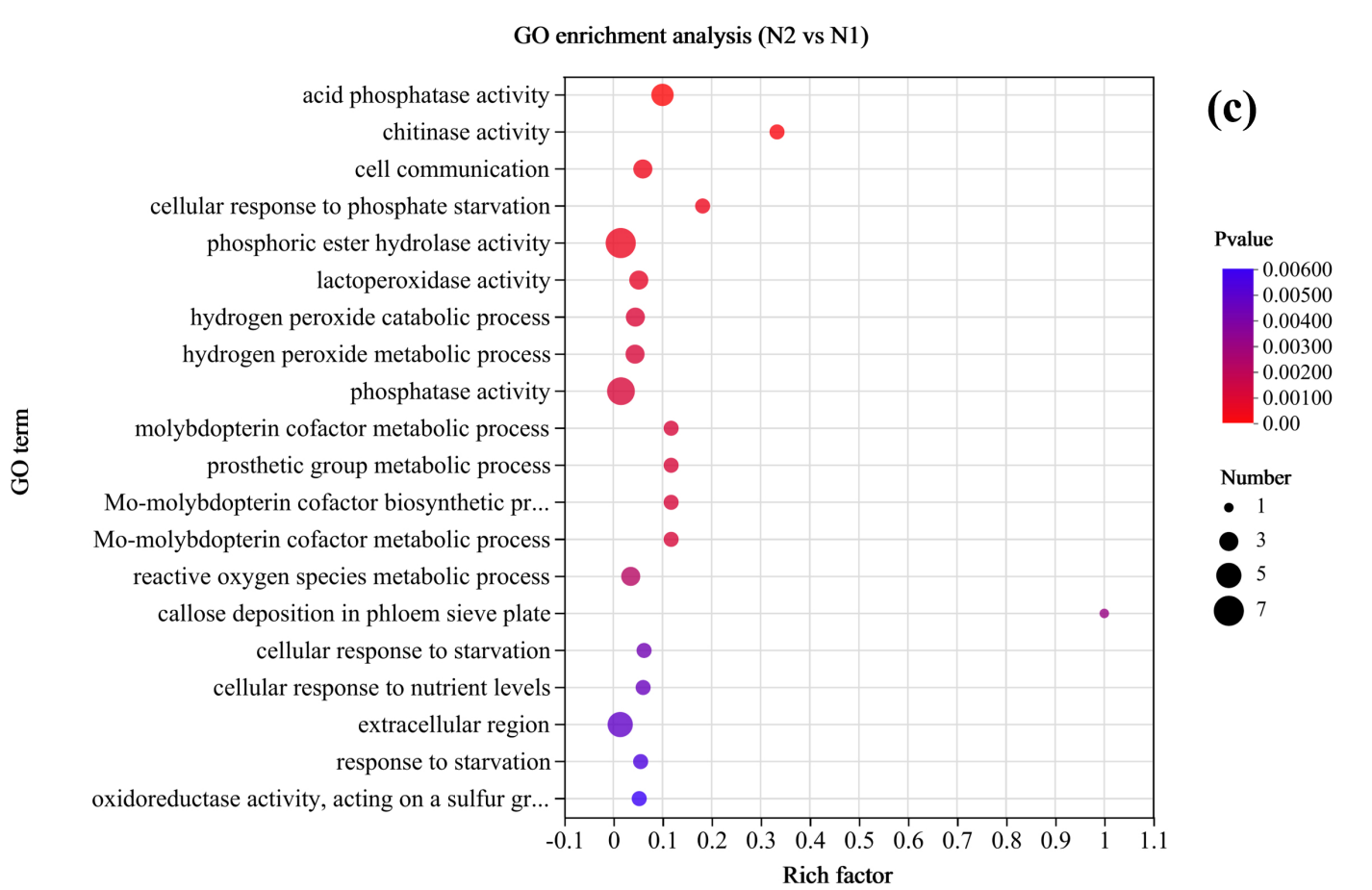

Supplement: Supplementary file 1 [file DataSheet1.zip › Supplementary files/Figure S3.pdf]

# KEGG enrichment analysis (N2 vs N1)

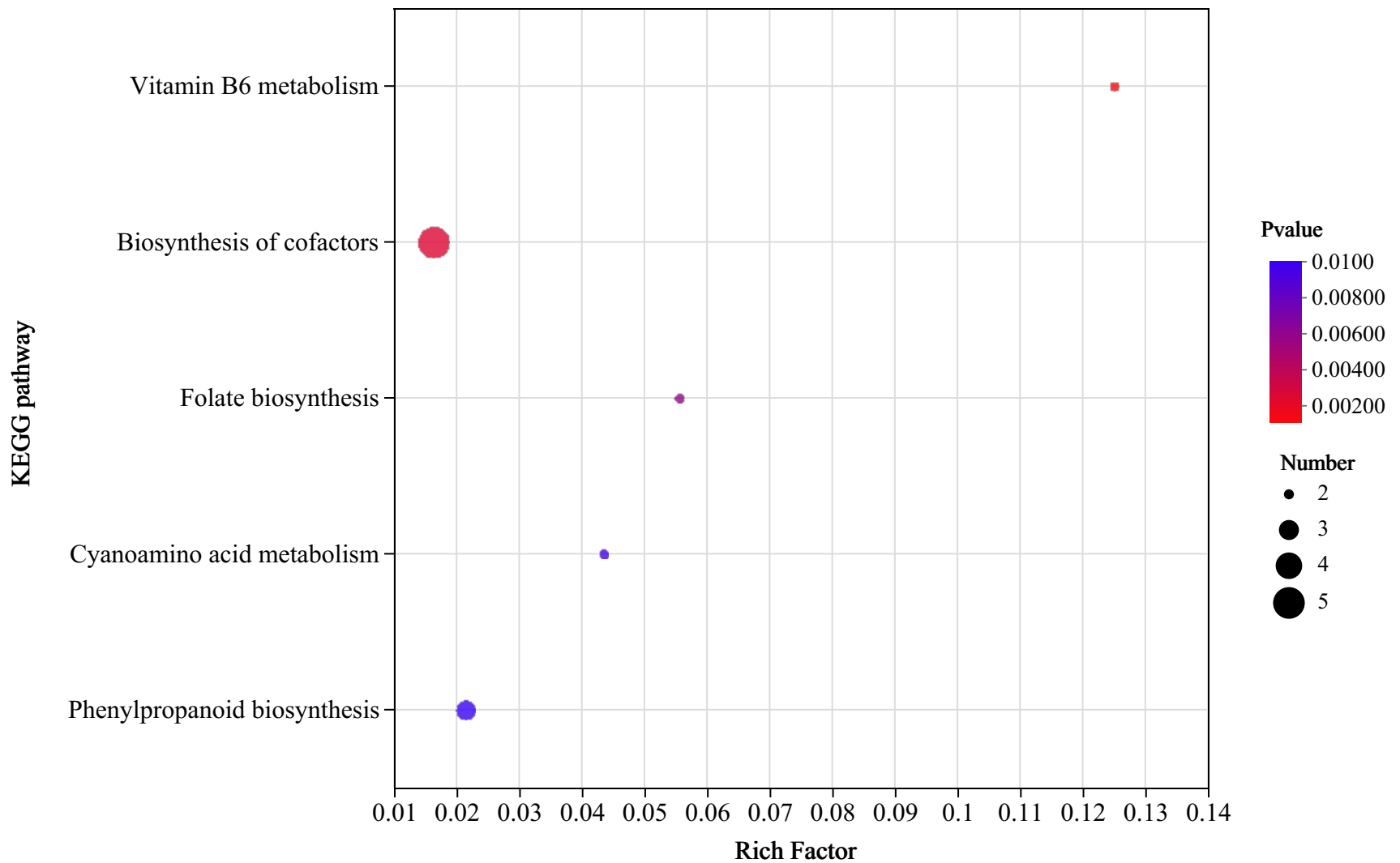

Supplement: Supplementary file 1 [file DataSheet1.zip › Supplementary files/Figure S4.pdf]

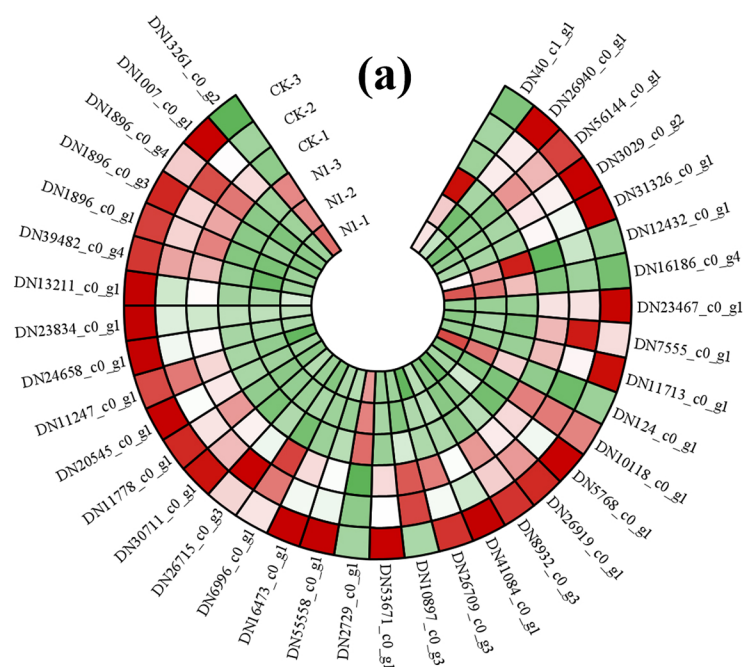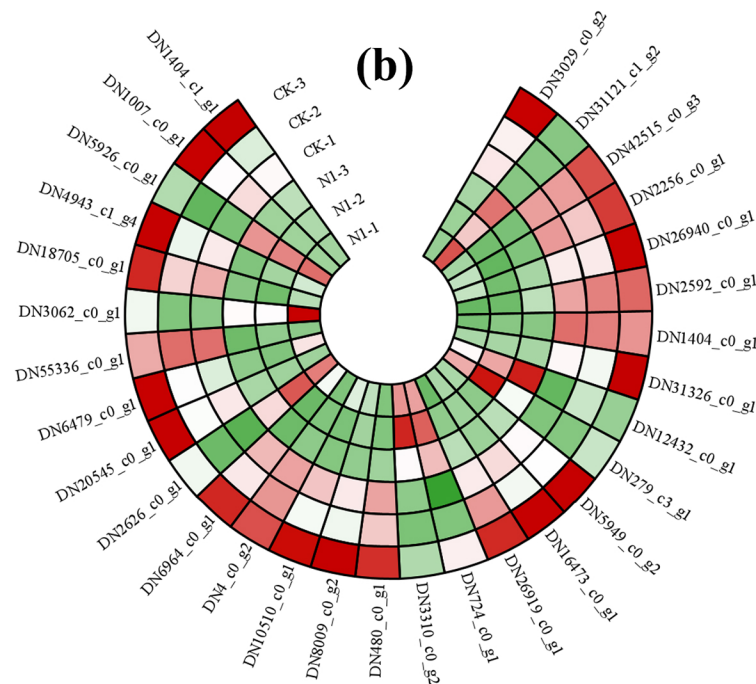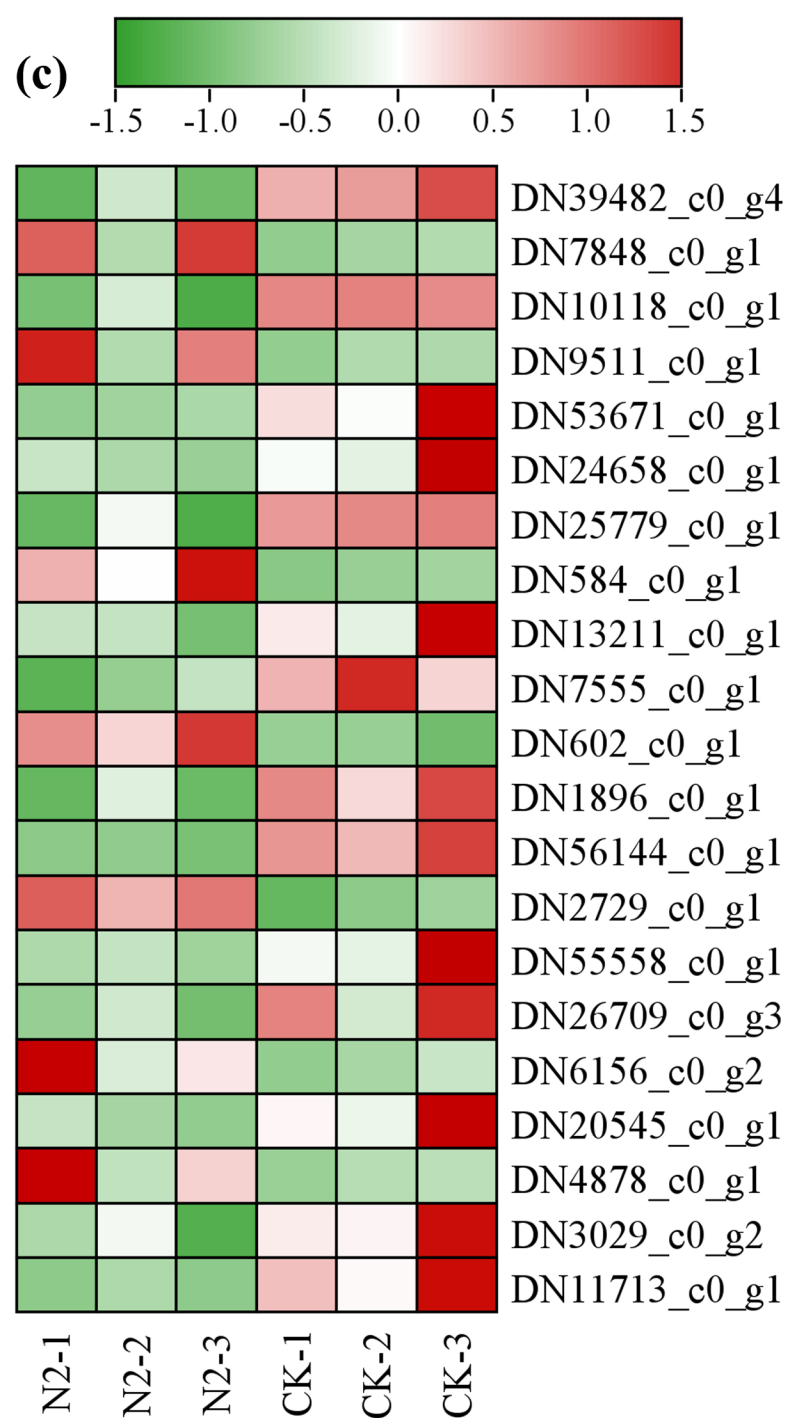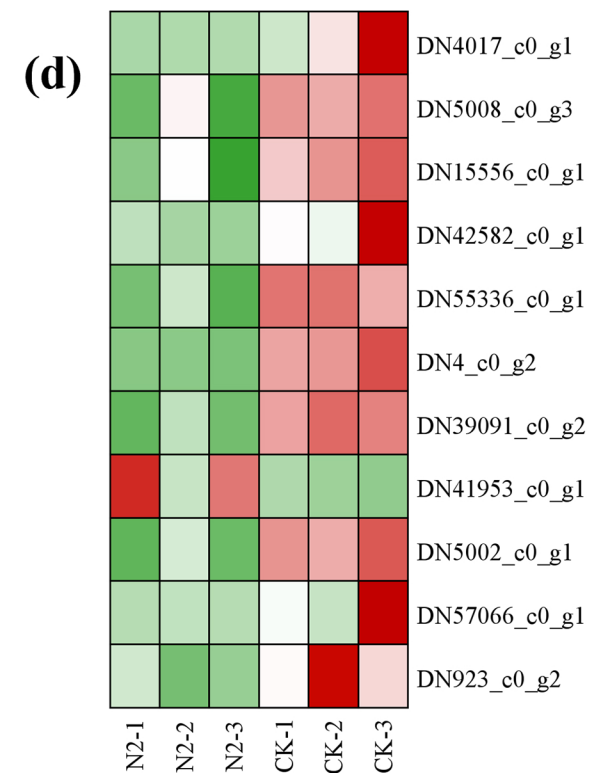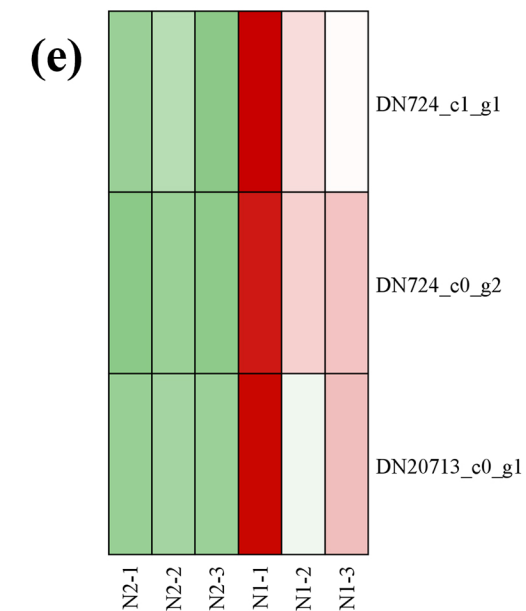

Supplement: Supplementary file 1 [file DataSheet1.zip › Supplementary files/Figure S5.pdf]

FLAVONOID BIOSYNTHESIS

(a)

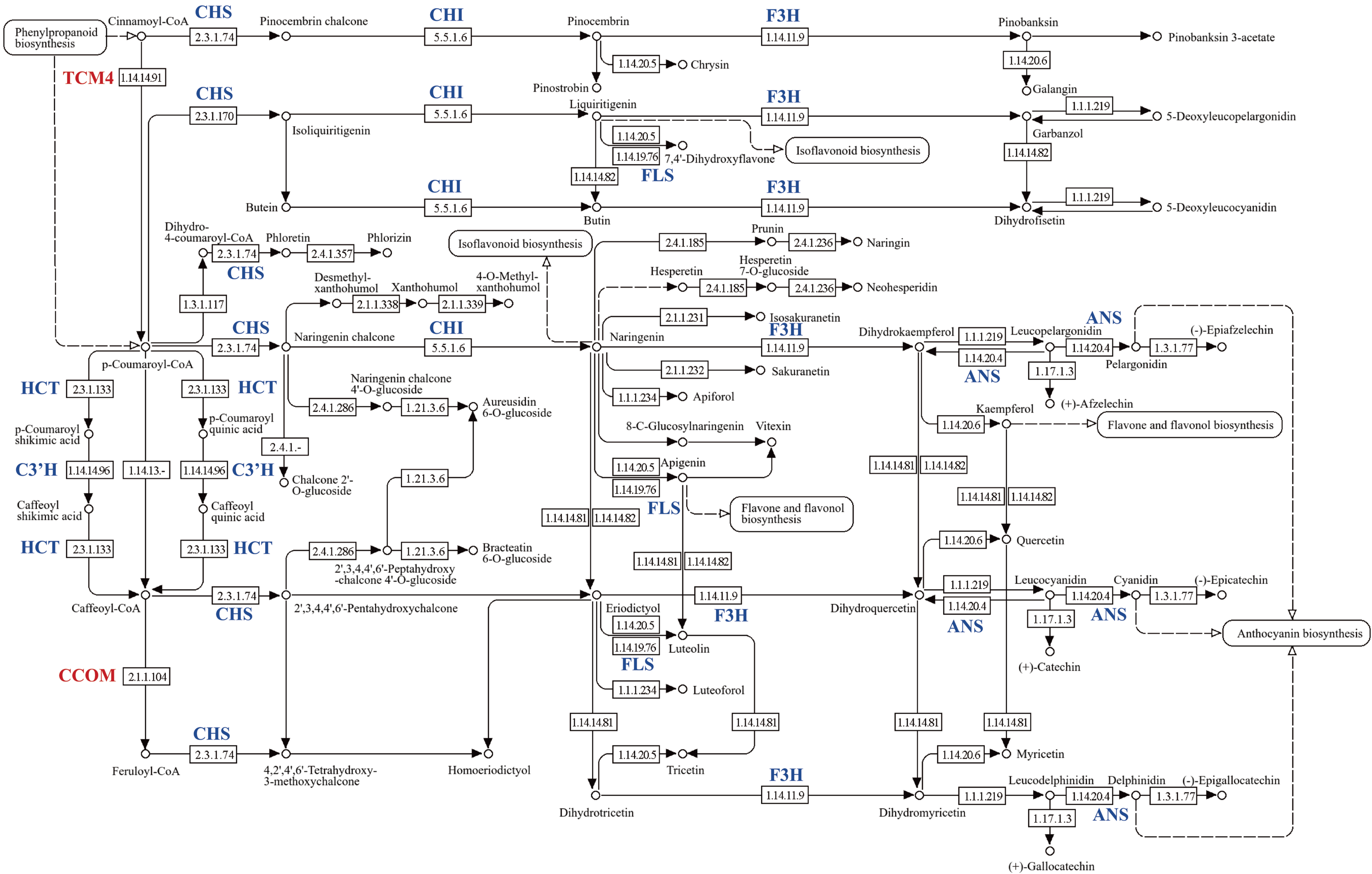

(b)

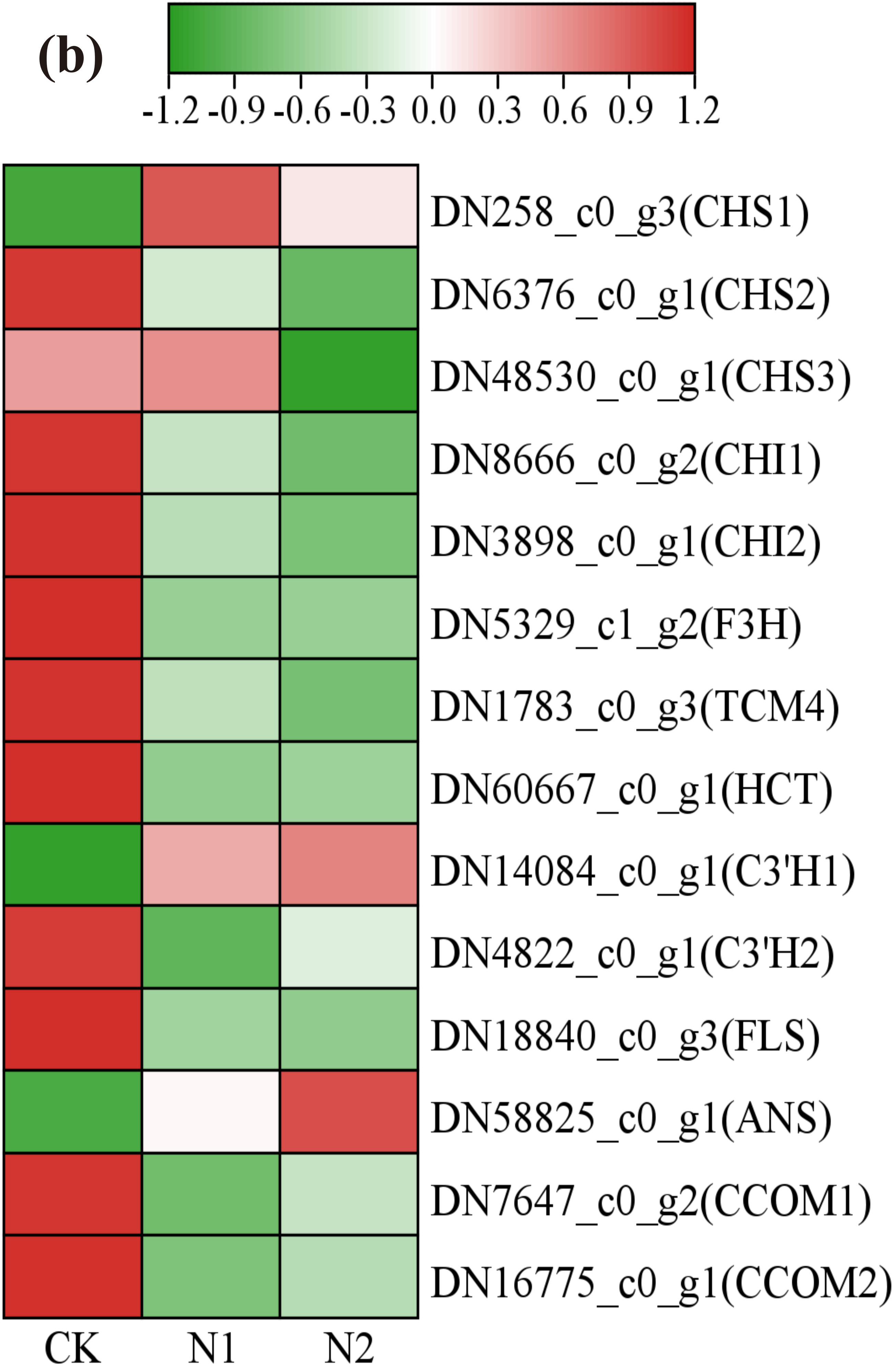

Supplement: Supplementary file 1 [file DataSheet1.zip › Supplementary files/Figure S6.pdf]
